# The *ΔfbpAΔsapM* candidate vaccine derived from *Mycobacterium tuberculosis* H37Rv is markedly immunogenic in macrophages and induces robust immunity to tuberculosis in mice

**DOI:** 10.3389/fimmu.2024.1321657

**Published:** 2024-06-21

**Authors:** Abhishek Mishra, Arshad Khan, Vipul Kumar Singh, Emily Glyde, Sankaralingam Saikolappan, Omar Garnica, Kishore Das, Raja Veerapandian, Subramanian Dhandayuthapani, Chinnaswamy Jagannath

**Affiliations:** ^1^ Department of Pathology and Genomic Medicine, Houston Methodist Research Institute, Weill-Cornell Medicine, Houston, TX, United States; ^2^ Department of Molecular and Translational Medicine, Texas Tech University Health Sciences Center El Paso, El Paso, TX, United States

**Keywords:** tuberculosis, vaccine, *Mtb*-derived, live vaccine, immunogenic, macrophages, mice

## Abstract

Tuberculosis (TB) remains a significant global health challenge, with approximately 1.5 million deaths per year. The Bacillus Calmette-Guérin (BCG) vaccine against TB is used in infants but shows variable protection. Here, we introduce a novel approach using a double gene knockout mutant (DKO) from wild-type *Mycobacterium tuberculosis* (*Mtb*) targeting *fbpA* and *sapM* genes. DKO exhibited enhanced anti-TB gene expression in mouse antigen-presenting cells, activating autophagy and inflammasomes. This heightened immune response improved *ex vivo* antigen presentation to T cells. Subcutaneous vaccination with DKO led to increased protection against TB in wild-type C57Bl/6 mice, surpassing the protection observed in caspase 1/11-deficient C57Bl/6 mice and highlighting the critical role of inflammasomes in TB protection. The DKO vaccine also generated stronger and longer-lasting protection than the BCG vaccine in C57Bl/6 mice, expanding both CD62L^-^CCR7^-^CD44^+/-^CD127^+^ effector T cells and CD62L^+^CCR7^+/-^CD44^+^CD127^+^ central memory T cells. These immune responses correlated with a substantial ≥ 1.7-log_10_ reduction in *Mtb* lung burden. The DKO vaccine represents a promising new approach for TB immunization that mediates protection through autophagy and inflammasome pathways.

## Introduction

Tuberculosis (TB) caused by *Mycobacterium tuberculosis* (*Mtb*) remains a major cause of death due to infections among children and adults with nearly 10.6 million cases and approximately 1.6 million deaths in 2021. Despite its widespread use, the Bacillus Calmette-Guérin (BCG) vaccine (administered to infants) exhibits limited efficacy against lung TB in children and TB in adults. Though BCG induces trained immunity against other pathogens (1) and provides better protection against extrapulmonary TB in infants, the need for improved TB vaccines has led to the exploration of many booster vaccines, including recombinant BCG vaccines, subunit protein, DNA-based vaccines, and virally vectored vaccines (2).

Live attenuated vaccines have emerged as promising candidates for longer-lasting immunity. However, only a few of these have entered clinical trials, including the recombinant *M. bovis*-derived BCG VPM1002, the *Mtb*-derived vaccine (*MTB*VAC), and *M. vaccae* (3). Efforts to revaccinate infants using BCG are also in progress (3), although this approach is contraindicated in HIV-I-infected individuals.

In our previous studies, we sought to develop a mechanism-based vaccine for enhancing BCG effectiveness. We demonstrated that the decreased efficacy of the BCG vaccine is likely due to its inability to undergo phagosome-lysosome (PL) fusion in antigen presenting cells (APCs), macrophages and dendritic cells, resulting in decreased *ex vivo* antigen presentation and CD4 T cell activation (4). We subsequently developed an autophagy-inducing first-generation recombinant BCG^85B^ and second-generation BCG^85BC5^ vaccine, which enhanced antigen processing in APCs *ex vivo*, induced robust T cell responses *in vivo*, and offered enhanced protection against aerosol-induced TB relative to BCG in mice (5).

Given the correlation between the efficacy of recombinant BCG and its ability to enhance antigen processing in APCs (5), we sought to develop similar vaccines using wild-type *Mtb*-H37Rv. Unlike BCG, wild-type Mtb possesses the RD1 region, which encodes ESAT-6 and CFP-10, along with nearly 90 open reading frames absent in BCG (6). This difference suggests that *Mtb*-derived attenuated vaccines may be more immunogenic due to the presence of immunodominant antigens. Given this, we generated single deletion mutants *fbpA*KO *(ΔfbpA)*, and *sap*MKO *(ΔsapM)* from wild-type *Mtb*-H37Rv (7, 8). *fbpAKO* demonstrated attenuated growth within mouse MΦs and human THP-1 MΦs, and emerged as a promising candidate vaccine in mice (**Supplementary Figure 1**) (8–10). Because the single gene mutant *fbpA*KO mutant was as effective as the BCG vaccine in protecting mice against aerosol-induced TB (9), we produced a *fbpA* and *sapM* double knockout mutant (*ΔfbpAΔsapM*; DKO) (8), which exhibited attenuated growth in mice similar to BCG and persisted at low numbers for up to 150 days following aerosol infection (**Supplementary Figure 1**) (8). In this study, we demonstrate that DKO induces robust gene expression in antigen-presenting cells, resulting in a hyper-immunogenic phenotype characterized by enhanced antigen processing and TH1 cytokine secretion through an autophagy and inflammasome-dependent mechanism. Collectively, our findings demonstrate that DKO offers superior protection against TB in mice relative to BCG Pasteur.

## Results

### The DKO mutant localizes to lysosomes through autophagy induction in macrophages

We generated single deletion mutants *fbpA*KO *(ΔfbpA)*, *sap*MKO *(ΔsapM)*, along with the double deletion ‘DKO’ mutant (*ΔfbpAΔsapM*), derived from *Mtb*-H37Rv (7, 8). The mutants exhibited attenuated growth within mouse MΦs and human THP-1 MΦs (**Supplementary Figures 1A, B**) (8). To create the DKO mutant, we deleted the *sapM* gene in the *fbpA* mutant, which resulted in attenuated growth comparable to that seen with BCG Pasteur over 150 days in C57Bl/6J mice (**Supplementary Figure 1C**) (8–10). *In vivo*, virulent *Mtb* predominantly localizes to MФs and uses various enzymes to evade and suppress immune responses (11). Therefore, we sought to determine whether the attenuation of *ΔfbpAΔsapM* growth could be attributable to its susceptibility to MФs-mediated killing. Activated MФs play a major role in *Mtb* elimination, primarily relying on IFN-γ activation to generate nitric oxide (NO) and reactive oxygen species (ROS). However, autophagy and PL fusion, mechanisms that deliver mycobacteria to lysosomes for degradation, operate innately. We hypothesized that DKO could be susceptible to these mechanisms.

**Figure 1:**
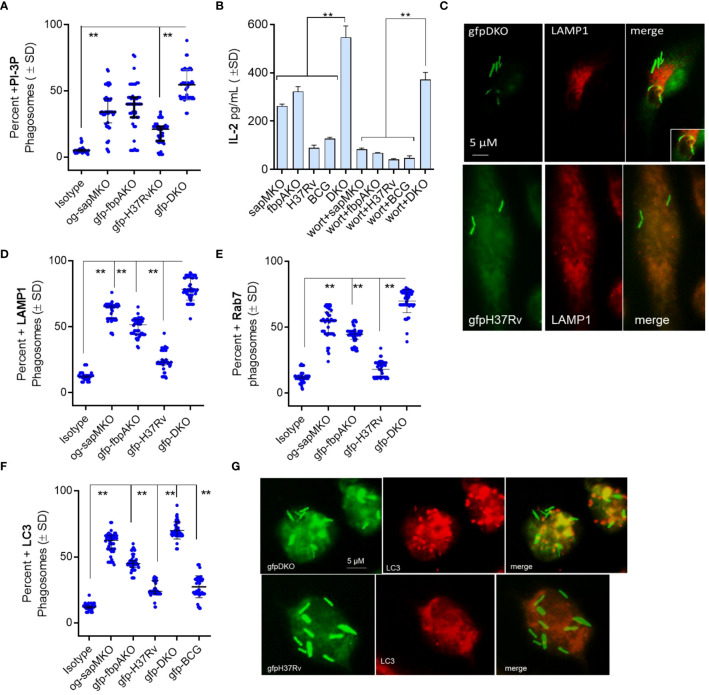
*Mycobacterium tuberculosis* (H37Rv)-derived *fbpA* and *sapM* double gene knockout (Δ*fbpA*Δ*sap*M; DKO) mutant undergoes phagosome-lysosome fusion in mouse macrophages. **(A)** C57Bl/6J-derived MΦs were infected with either Oregon green (*og*)-labeled *sapM*KO (*og-sapM*KO), *gfp*-labeled *fbpA*KO, *gfp*-*Mtb*-H37Rv, or *gfp*-DKO (MOI=1). After 18 h of incubation, cells were fixed and stained with either an isotype antibody or an antibody targeting phosphatidiyl-inositol-3 phosphate (PI-3P). Counterstaining was performed with Alexa-fluor 590 conjugates. Confocal fluorescence microscopy (CFM) was used to quantify the number of colocalizing mycobacterial phagosomes among 30 MΦs per chamber in triplicates. **(B)** MΦs infected with mycobacteria were treated with the PI-3 kinase inhibitor wortmannin (1 µM), then washed and overlaid with BB7 T cells for *in vitro* antigen presentation. **(C–E)** Phagosomes, prepared as in panel **(A)**, were stained for Lysosome-associated membrane protein-1 (LAMP1; panel c) and Rab7 (images not shown) followed by quantitation of colocalization. **(F, G)** CD11c^+^ dendritic cells (DCs) purified from the bone marrow of wt-C57Bl/6J mice were infected with fluorescent mycobacteria. Cells were then labeled using a monoclonal antibody against microtubule-associated light chain-3 (LC3), a canonical marker of autophagy. CFM analysis displayed phagosomes colocalizing with LC3 (**p< 0.009, two-tailed *t-*test).

First, we evaluated the level of phosphatidyl-inositol-3 phosphate (PI-3P) on DKO phagosomes relative to *fbpA*KO, *sapM*KO, and *Mtb-H37Rv*, as DKO lacks the *sapM* phosphatase that dephosphorylates PI-3P on the phagosome membrane (12). We infected MФs with *gfp*-*Mtb*-H37Rv, Oregon-green labeled *sapM*KO (og-*sapM*KO), *gfp*-*fbpA*KO or *gfp-*DKO, followed by incubation with a PI-3P-specific antibody and conjugates and then confocal fluorescent microscopy imaging (CFM) (5). *gfp-*DKO phagosomes were highly enriched for PI-3P compared to other mycobacteria (**Figure 1A**). We have previously demonstrated that BCG and *Mtb*-infected MФs present the Antigen85B-derived P25 epitope to BB7 CD 4 T cells, eliciting an IL-2 response using a protocol established by Harding et al. (4, 13, 14). To assess the importance of PI-3P-Rab7-dependent lysosomal fusion in antigen presentation (15), we treated MФs with wortmannin, a specific PI-3 kinase inhibitor, or control before infecting them with mycobacteria and assessing antigen presentation to BB7 CD4 T cells. **Figure 1B** shows that wortmannin significantly inhibited antigen presentation in mycobacteria-infected MФs compared to DMSO vehicle control diluted in PBS. To validate PL fusion, we performed immunostaining for LAMP1 and Rab7 markers on MФs phagosomes; **Figures 1C–E** shows that *gfp-*DKO phagosomes were enriched for these lysosomal markers.

RAB*-* and SNARE-dependent sorting of mycobacterial phagosomes to lysosomes is well characterized (16–19), but mycobacteria can also be delivered to lysosomes via autophagy (20, 21). Autophagy is a homeostatic mechanism of mammalian cells responsible for delivering misfolded proteins, damaged organelles, and cytosol into autophagolysosomes (APLs) for degradation. During autophagy, many intracellular pathogens are also internalized into autophagosomes, which are then transported into APLs for degradation through macroautophagy (22). Because *sapM* is deleted in DKO and PI3-P is enriched on DKO phagosomes (**Figure 1A**), we hypothesized that the DKO mutant is sorted to APLs through autophagy. To test this, we infected MФs with fluorescently tagged mycobacteria and performed immunostaining using an antibody against microtubule-associated light chain-3 (LC3), a canonical marker of autophagosomes, followed by CFM analysis. **Figures 1F, G** shows that *gfp-*DKO strongly colocalized with LC3 compared to phagosomes containing other mycobacteria.

### The DKO mutant enhances expression of genes regulating autophagy induction in macrophages

**Figure 2:**
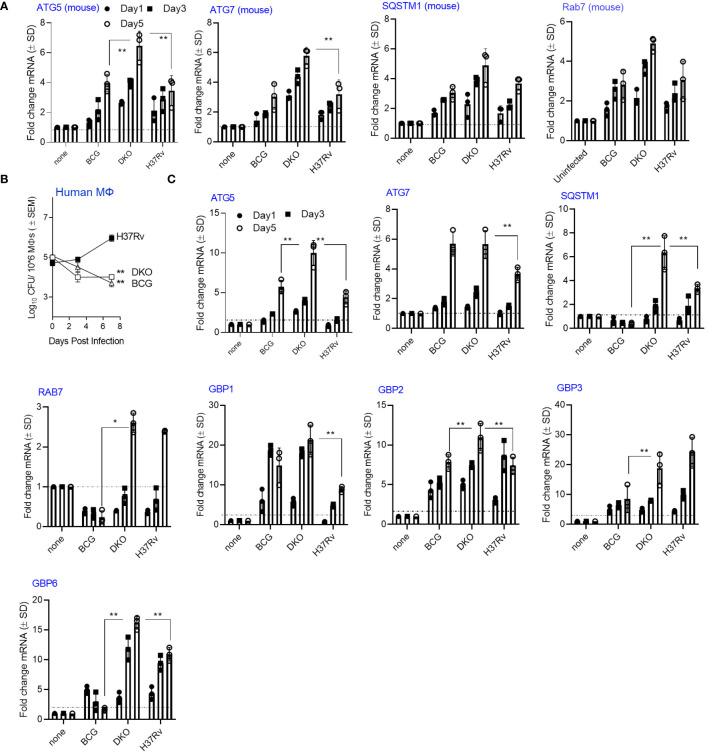
*Mycobacterium tuberculosis* (H37Rv)-derived *fbpA* and *sapM* double gene knockout mutant (Δ*fbpA*Δ*sap*M; DKO) upregulates genes associated with autophagy regulation in macrophages. **(A)** C57Bl/6J bone marrow-derived CD14+ MФs (pooled from 3 mice) were infected with DKO, *M. bovis* BCG Pasteur, or *Mtb*-H37Rv (MOI:1) for 4 h, washed, and then incubated. On the indicated days, Trizol lysates were collected and analyzed using qRT-PCR for mRNA of the specified genes (**p< 0.006, two-tailed t-test). The growth curve for the mycobacteria is shown in **Supplementary Figure 1**. **(B)** Human MФs were infected with DKO, *M. bovis* BCG Pasteur, or *Mtb*-H37Rv (MOI:1) for 4 h, washed, and then incubated. On the indicated days, lysates were plated on 7H11 agar for growth analysis (** p< 0.004 one-way ANOVA). **(C)** Trizol lysates from days 1, 3, and 5 from panel **(A)** were analyzed using qRT-PCR for mRNA of the specified genes (*, **p< 0.009, two-tailed t-test). .

In our recent study, we observed upregulation of autophagy-regulating genes in the lungs of mice and in MФs *ex vivo* following administration of an adenovirus-based nasal TB vaccine (23). We performed a kinetic assay using RT-PCR in DKO-infected MФs, as virulent *Mtb* can downregulate autophagy through a *sapM-*dependent mechanism (24, 25). First, we tested CD14 bead-purified MФs from C57Bl/6J mice for gene expression. Next, we infected MФs derived from healthy donors (n=3) with DKO, BCG, or *Mtb*-H37Rv. We analyzed growth curves over 7 days, and on days 1, 3, and 5, we analyzed the expression of autophagy-controlling genes using qRT-PCR. Autophagy is regulated by autophagy-regulating genes, including *ATG5 and ATG7*, while *SQSTM1* (p62) serves as an autophagosome biogenesis substrate (26). The Rab7 protein, which is involved in endosome sorting, mediates PL fusion and APL fusion (27, 28). **Figure 2A** shows that DKO induced markedly higher expression of four autophagy-regulating genes (*ATG5/7, SQSTM1* and *Rab7*) compared to *Mtb* H37Rv and BCG vaccine, a finding that correlated with its attenuated growth (**Supplementary Figure 1**). **Figure 2B** shows that both BCG and DKO exhibited growth attenuation in human MФs compared to *Mtb*-H37Rv. In these human MФs, DKO induced a time-dependent increase in gene expression for *ATG5/7, SQSTM1*, and *Rab7* compared to *Mtb*-H37Rv or BCG vaccine (**Figure 2C**). Notably, DKO also increased the expression of genes encoding Guanylate binding proteins (GBPs) in human MФs. GBPs are IFN-γ-inducible proteins that regulate host defense via phox proteins of the phagocyte oxidase, antimicrobial peptides, and autophagy effectors (29). Conversely, we found no difference in GBP gene expression in mouse MФs. Because we used naïve mouse and human MФs for mycobacterial infection, these gene expression findings highlight the increased immunogenicity of DKO relative to *Mtb* H37Rv, emphasizing its capacity to activate autophagy instead of suppressing it (25, 30).

### DKO candidate vaccine induces autophagy in DCs and MΦs and enhances *in vitro* antigen presentation to CD4 T cells

**Figure 3:**
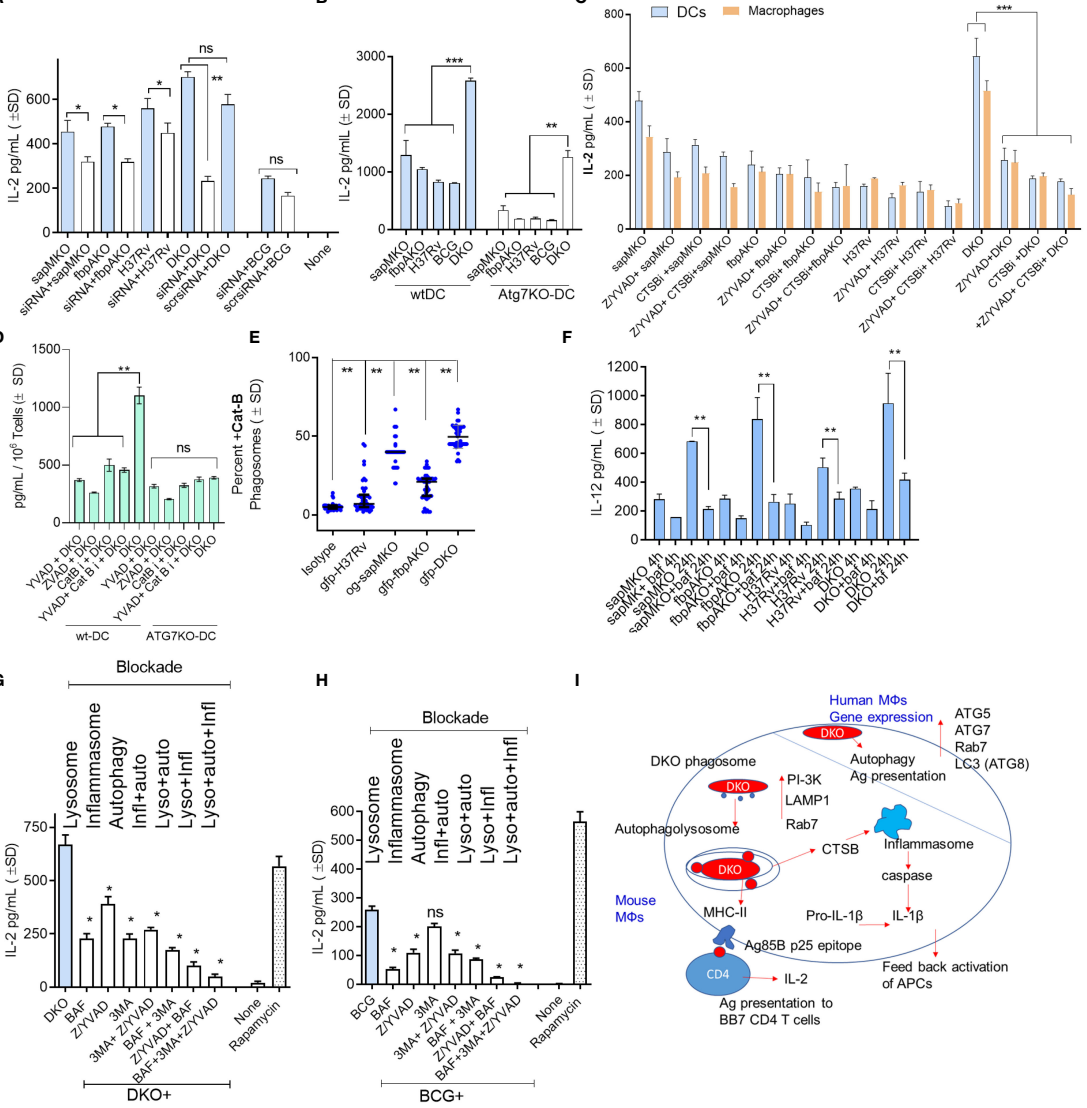
DKO mutant induces autophagy- and inflammasome-dependent antigen presentation in dendritic cells. **(A)** wt-DCs were either treated with siRNA targeting beclin1 (*ATG*6) or left untreated, followed by infection with mycobacteria (MOI=1). Subsequent T cell overlay was used to evaluate antigen presentation (*, **p< 0.01, two-tailed t*-*test). **(B)** wt-APCs sourced from wild-type C57Bl/6J and *Atg*7DC-KO mice were infected with mycobacteria (MOI=1). Antigen presentation was measured 18 h post-infection (**, ***p< 0.007 two-tailed t*-*test). **(C)** wt-APCs were either treated with Cathepsin-B inhibitor (CA 074; 1 µM), a mix of ZVAD and YVAD (40 µM each) or left untreated, followed by infection with mycobacteria (MOI-1; 4 h). IL-2 levels in supernatants collected 4 h post-overlay were used as a measure of antigen presentation (***p< 0.008 two-tailed t*-*test). **(D)** DCs from wt and *Atg*7DC-KO mice were infected with DKO, treated with ZVAD/YVAD and CA 0174, and assessed for antigen presentation (** p< 0.006 two-tailed t*-*test). **(E)** DCs were infected with fluorescently labeled mycobacteria and immunostained using antibodies specific to CTSB. A colocalization assay using CFM was then performed (**p< 0.01, two-tailed t*-*test). **(F)** CD11c^+^ dendritic cells (DCs) purified from the bone marrow of wt-C57Bl/6J mice were treated with the vATPase inhibitor bafilomycin (100 nM), followed by infection with mycobacteria and *in vitro* antigen presentation. (**p< 0.009 two-tailed t-test). **(G, H)** wt*-*DCs were either treated with bafilomycin (100 nM), 40 µM each of ZVAD and YVAD, 50 µM of 3-methyladenine, combinations thereof as indicated, or left untreated. Cells were then infected with either DKO **(G)** or BCG **(H)**. Rapamycin (1 µg/mL) which induces autophagy was used as a positive control. Antigen presentation was evaluated relative to DKO using IL-2 levels in supernatants collected 18 h post-infection (*p< 0.01 ** two-tailed t*-*test; ns, not significant.). Cell viability was confirmed to be >90% using trypan blue. **(I)** A schematic illustrating the proposed mechanism for antigen processing of DKO in APCs. For all panels, results from one representative experiment out of two or three similar experiments are shown.

In our prior studies, we reported a correlation between the ability of BCG and recombinant BCG vaccines to confer protection against TB in mice and PL fusion, either through the Rab7 and SNARE-dependent phagosome maturation pathway or *ATG*-dependent autophagy pathway (4, 9, 10). (5, 31) Indeed, lysosomal degradation of the vaccine is essential for generating MHC-II-dependent peptide epitopes (32). Therefore, we sought to determine whether lysosomal localization renders DKO-infected APCs more immunogenic. Using an *ex vivo* model from Harding et al., we demonstrated that mycobacteria-infected mouse APCs rapidly present Ag85B-derived P25 epitope to BB7 CD4 T cells *in vitro (* 4, 13, 14 *).* Because autophagy regulates MHC-II-dependent mycobacterial antigen presentation in DCs (5), we subjected *wt*-DCs to siRNA-mediated beclin1 (*ATG6*) knockdown followed by infection with non-labeled mycobacteria and antigen presentation assays. **Figure 3A** shows that autophagy knockdown significantly reduced antigen presentation in DCs overlaid with Ag85Bp25 specific CD4 T cells when they were infected with *sapM*KO, *fbpA*KO, *Mtb* H3Rv, and DKO, but not in BCG-infected DCs. To validate specificity, we assessed bone marrow-derived DCs from wild-type C57Bl/6 mice and C57Bl/6 mice with a DC-specific deletion of *ATG7* (*ATG7*KO-DC) (kindly provided by Dr. Jim Wang, Houston Methodist Research Institute, Houston TX) (33). **Figure 3B** shows that mycobacteria-infected *ATG7*KO-DCs overlaid with Ag85Bp25 specific CD4 T cells also showed reduced antigen presentation compared to wild-type DCs, confirming the key role of autophagy. Notably, DKO-infected DCs continued to present antigens.

Previously, we observed that the DKO mutant induced elevated levels of IL-1β in mouse APCs (10). IL-1β plays a key role in protection against TB and increases antigen presentation in mycobacteria-infected MФs (34). We hypothesized that DKO-induced autophagy and inflammasome activation might synergize to enhance antigen presentation (35). For example, Cathepsin-B (CTSB) might leak from lysosomes containing DKO and activate inflammasomes, releasing IL-1β (36, 37). To test this hypothesis we treated wild-type APCs with the pan-caspase specific inhibitor ZVAD-fmk (ZVAD) or caspase-1 specific inhibitor YVAD-fmk (YVAD), with or without CA-074, an inhibitor of CTSB (CTSBi), followed by antigen presentation assays. Because antigen presentation to T cells is rapid, we measured IL-2 at 4 h post-infection and 4 h post-overlay of T cells. **Figure 3C** reveals an interesting finding: caspase and CTSB blockade reduced antigen presentation by DKO-infected APCs but not by *Mtb* or BCG-infected DCs. Notably, both caspase and CTSB blockade were effective in reducing antigen presentation in wild-type DCs but not in *ATG7*KO-DCs (**Figure 3D**). This suggests a connection between inflammasome and autophagy in DKO-infected APCs, likely mediated by CTSB leaked from lysosomes. Indeed, CFM studies of DCs confirmed that DKO phagosomes were enriched for CTSB. (**Figure 3E**). To further validate that DKO triggers both inflammasome and autophagy during antigen processing, we successively treated wild-type DCs with inhibitors of lysosomal acidification (bafilomycin), autophagy (3-methyladenine) (4), and inflammasome, either alone or in combination, followed by infection with DKO or BCG and antigen presentation assays. As *Mtb* H37Rv cannot be used as a vaccine, it was not tested. **Figures 3F-H** indicate that antigen presentation by DKO and BCG-infected DCs was nearly abolished when all three mechanisms were concurrently inhibited. Remarkably, unlike DKO-infected DCs, antigen presentation by BCG-infected DCs remained unaffected by autophagy inhibition (**Figure 3I**). DKO-infected DCs, therefore, show unique antigen processing pathways.

### *Mtb*-H37Rv-derived single *(sap*MKO*, fbpA*KO) and DKO mutants induce a differential IL-1β and T_H_1 cytokine response in APCs from caspase-1/11-deficient mice

**Figure 4:**
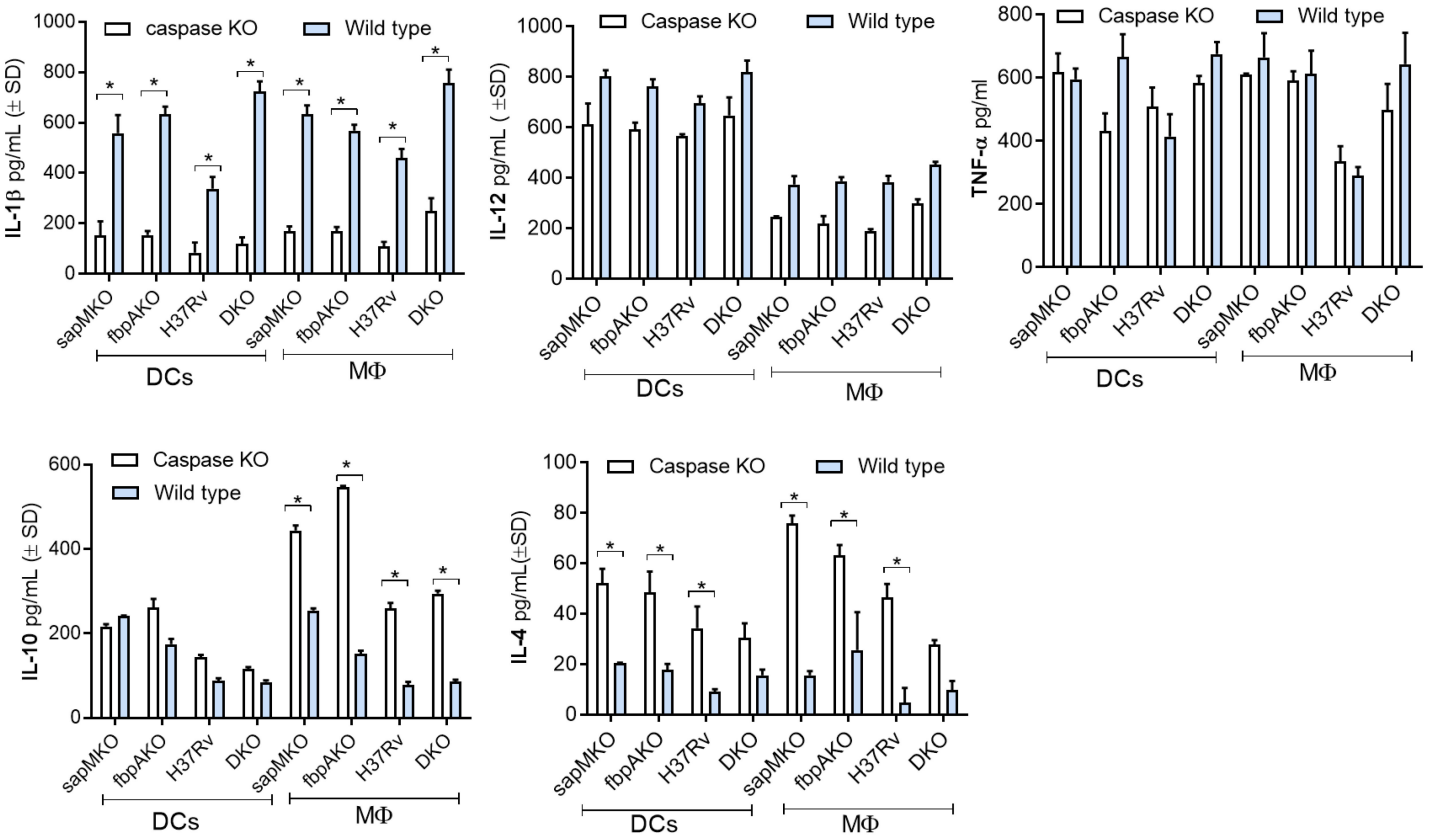
DKO mutant induces caspase-dependent IL-1β from mouse antigen-presenting cells. Bone marrow-derived macrophages (MФs) and dendritic cells (DCs) (collectively, antigen-presenting cells; APCs) from wild-type C57Bl/6J or C57Bl/6J-background caspase 1/11-deficient mice were infected with *Mtb* or its mutants (MOI=1). Supernatants were collected 18 h post-infection and tested for IL-1β and indicated TH1 cytokines using sandwich ELISA. A comparison of IL-1β secretion after caspase blockade in DKO-infected APCs versus *Mtb* mutants and the BCG vaccine is shown in **Supplementary Figure 2**. *p< 0.01, two-tailed t-test.

TB vaccines induce pro-inflammatory cytokines from APCs and activate T cells. Given the significance of IL-1β as a key cytokine for protection against TB, and the ability of *Mtb* to evade inflammasome-dependent surveillance mechanisms in MФs (38), first we used wild-type C57Bl/6J mouse bone marrow-derived APCs for infection experiments, followed by cytokine assays. To ensure specificity, we then used APCs from C57Bl/6 background capsase-1/11 KO mice (kindly provided by Dr. Dmitry Shayakhmetov, Emory University). DKO enhanced the secretion of IL-1β in wild-type DCs and MΦs compared to *sapM*KO, *fbpA*KO, or *Mtb*-H37Rv. Importantly, APCs derived from caspase-1/11 KO mice showed reduced levels of IL-1β but maintained comparable levels of (T_H_1-driving) pro-inflammatory IL-12 and TNF-α. Conversely, anti-inflammatory cytokines IL-10 and IL-4 were increased among caspase-1/11 KO-derived APCs (**Figure 4**). In additional studies, we induced pharmacological blockade of caspase in C57Bl/6J bone marrow-derived APCs using ZVAD and YVAD. **Supplementary Figure 2** illustrates that DKO-induced IL-1β was dependent on the caspase pathway in both MФs and DCs. In contrast, *Mtb*-induced IL-1β was caspase-dependent only in DCs. This distinction suggests that the DKO mutant differs in its ability to activate the inflammasome in mouse APCs compared to *Mtb* (39).

### Enhanced caspase-dependent *ex vivo* antigen presentation to CD4 T cells partially protects mice against tuberculosis in the *Mtb*-H37Rv-derived DKO mutant

**Figure 5:**
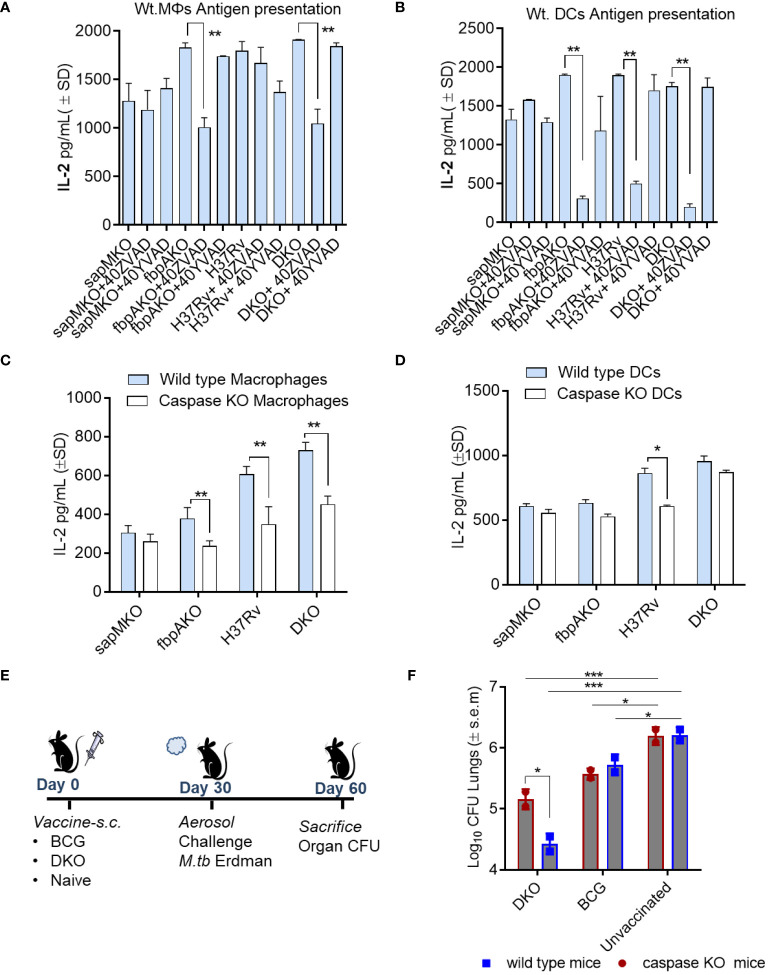
DKO mutant induces better antigen presentation in mouse APCs and protects mice against tuberculosis partially dependent upon IL-1β. **(A, B)** APCs derived from wt-C57Bl/6 mice were pre-treated with or without ZVAD-fml (pan-caspase specific inhibitor) and YVAD-fmk (caspase-1 specific inhibitor; 40 µM each) (**Supplementary Figure 4**). Following treatment, APCs were infected with *Mtb* or its mutants (MOI=1). Four hours after infection, washed APCs were overlaid with Ag85B-p25 epitope-specific BB7 hybridoma CD4 T cells. Supernatants were collected at 18 h and tested for IL-2 using ELISA. **(C, D)** APCs from wild-type C57Bl/6 or caspase-1/11-deficient mice were infected with *Mtb* or its mutants, followed by an antigen presentation assay (panel a-d; *p< 0.01, **p< 0.007 one-way ANOVA with Tukey’s *post hoc* test; results from 1 of 2 similar experiments with triplicates shown). **(E, F)** wild-type C57Bl/6 (n=3 per group) and caspase-1/11 KO mice (n=3 per group) were vaccinated with DKO or BCG Pasteur (10*6 CFU/mouse, s.c.), and then aerosol-challenged with *Mtb*-Erdman (tagged with acriflavine resistance gene). Post-challenge, mice were sacrificed, and *Mtb* CFU counts from lung homogenates were determined using 7H11 agar containing acriflavine (DKO vs. Erdman)(*p< 0.01; ***p< 0.007, two-way ANOVA).

Inflammasome and caspase-derived IL-1β plays a major role during the processing of intracellular pathogens (40). IL-1β also induces monocyte to MΦ differentiation, thereby enhancing antigen presentation (34). Notably, inflammasomes can affect both MHC-I and MHC-II-dependent antigen presentation (41, 42). Further, many immunogenic antigens of *Mtb* seem to drive DC maturation, leading to cytokine production, including IL-1β (42–47). We therefore sought to determine whether DKO, in addition to promoting TH1 cytokine secretion, increases the ability of mouse APCs to present mycobacterial antigens to T cells, rendering them more immunogenic. In an *in vitro* antigen presentation assay, mouse APCs rapidly presented an Ag85B-derived p25 epitope to BB7 hybridoma CD4 T cells, leading to secretion of IL-2 from T cells (14, 48). This assay serves as a predictive measure of the immunogenicity of *Mtb* mutants and BCG vaccine strains both *ex vivo* and *in vivo (* 4, 5, 14, 48 *).* (5, 31) APCs were treated with ZVAD or YVAD to block caspases, followed by infection and overlay with BB7 T cells in the absence of drugs. **Figures 5A, B** shows that ZVAD was able to inhibit DKO induced antigen presentation in both wt. MΦs and DCs, whereas *Mtb* H37Rv induced antigen presentation was inhibited only in DCs. Further, MΦs and to some extent DCs from Caspase KO mice showed reduced antigen presentation after mycobacterial infection. These data suggest that DKO and Mtb H37Rv affect the ability of APCs to present antigen to T cells (**Figures 5C, D**).

To determine whether caspase regulated IL-1β affected immunogenicity of the DKO vaccine *in vivo*, Caspase1/11 KO mice were then vaccinated and challenged with Mtb Erdman (**Figure 5E**). Because *Mtb* attains ~6-log_10_ growth over 4 weeks in C57Bl/6 mice, a decrease in the colony counts of *Mtb* (CFUs) in the lungs of vaccinated mice indicates protection. DKO generated better protection for the wild-type mice (~1.7-log_10_ reduction in CFUs) compared to a reduced level of protection (0.8-log_10_ reduction) in caspase KO mice (**Figure 5F**). However, the BCG vaccine exhibited similar levels of protection in both wild-type and caspase-1/11 KO mice. These data suggest that inflammasome-generated caspases and IL-1β likely contribute to DKO vaccine-induced protection against TB in mice.

### DKO vaccine induces robust protection against primary and reinfection tuberculosis in mice

**Figure 6:**
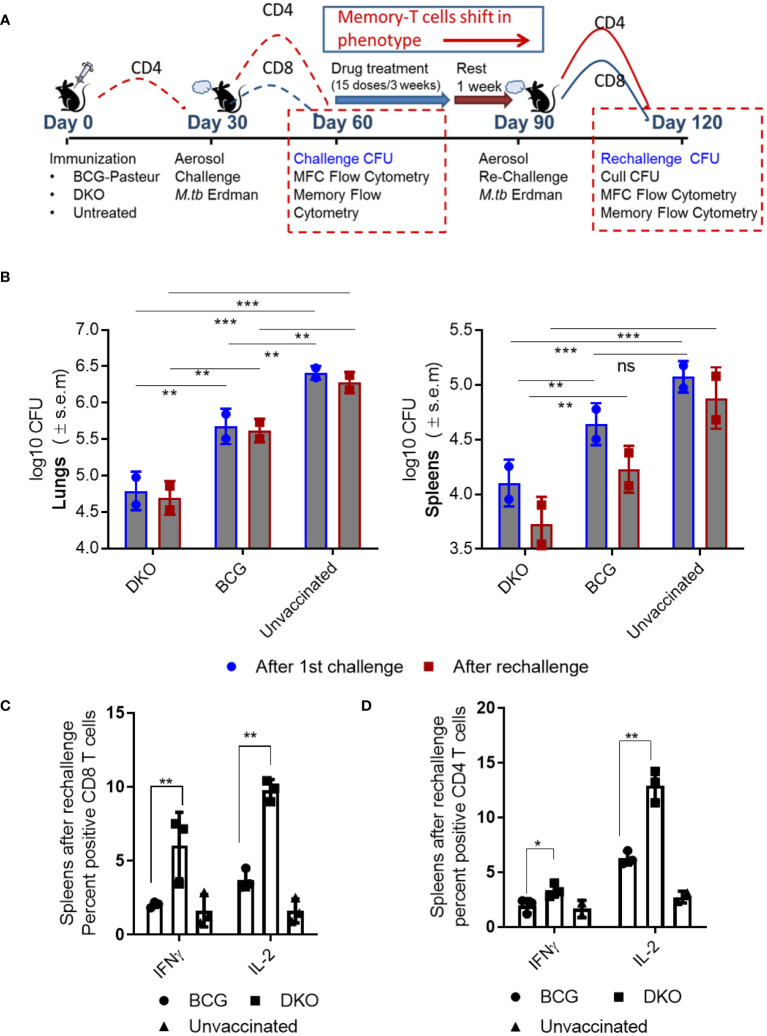
DKO vaccine induces robust protection against primary aerosol challenge and rechallenge with *Mycobacterium tuberculosis* Erdman in mice. **(A)** Schematic representation of vaccine-induced short-term (day 60) and long-term (day 120) protection model. C57Bl/6J mice (4–6 weeks old, male and female) were subcutaneously vaccinated with one dose of 10*6 CFU of BCG and DKO or left untreated. One group was aerosol-challenged using 100 CFU of *Mtb*-Erdman in a Glas-Col chamber and sacrificed on day 60 for *Mtb* CFU and T cell profile examination. The second group received a three-week treatment with a mix of isoniazid and rifampicin by gavage, followed by a 10-day rest period (during which organs showed no *Mtb* growth on 7H11 agar). Mice were then aerosol re-challenged using *Mtb*-Erdman and sacrificed on day 120. **(B)** Log_10_ reduction in *Mtb*-Erdman counts for the vaccine groups post-challenge and re-challenge. Data represent mean values (n=5 per group; p** 0.007; p *** 0.005, two-way ANOVA with Dunnett’s *post hoc* test). Results shown are from one of two similar independent experiments. **(C, D)** Spleens of mice were collected after necropsy on day 120 and stained for intracellular IFN-γ and IL-2 and analyzed using flow cytometry (*, **p< 0.01 two-tailed t test; ns, not significant).

Given the encouraging results suggesting that DKO is highly immunogenic in APCs, we sought to compare its efficacy with that of BCG in protecting mice against TB. Because *Mtb* can re-infect humans in endemic areas, we evaluated the efficacy of the DKO vaccine using two models. First, we examined its performance as a primary vaccine relative to BCG (**Figure 6A**; NIH challenge model). Subsequently, following drug-mediated clearance of both the vaccine and *Mtb* (verified by the absence of growth in post-treatment organs), we subjected mice to a low-dose *Mtb* re-challenge to evaluate the ability of DKO to protect against reinfection (**Figure 6A**, re-challenge model). In both models, we evaluated CFU counts and T cell responses.

*Efficacy of DKO vaccine in mice after a single dose Mtb challenge.* Vaccinated mice were rested for 30 days, and aerosol-challenged with a low dose of *Mtb*-Erdman, followed by necropsy on day 60 (**Figure 6A**). In this model, DKO outperformed BCG as a primary vaccine, generating >1.7 log_10_ protection in the lungs and ~0.9- log_10_ in the spleens compared to BCG (**Figure 6B**).

*Efficacy of DKO vaccine in mice following reinfection with Mtb.* Because BCG-induced protection in mice diminishes over time and offers limited protection against *Mtb* reinfection in mice (49), various reinfection models have explored the impact of repeated infections or vaccination on subsequent TB outcomes (50–52). We adapted a reinfection model wherein mice vaccinated and challenged (as described in **Figure 6A**) were cleared of the vaccines and *Mtb* using isoniazid (INH) and rifampin therapy, followed by a 10-day rest period when organs showed no *Mtb* growth on 7H11 agar. These mice were then re-challenged with aerosolized *Mtb*-Erdman. We hypothesized that T_EM_ generated during primary vaccination would transition into T_CM_ after chemotherapy and rest, thereby maintaining protection (49). **Figure 6B** shows that DKO was more efficient than BCG in reducing the *Mtb* burden in both the lungs and spleens, even after mice were reinfected. Notably, when C57Bl/6J mice are revaccinated with BCG (homologous booster) and then challenged, they fail to protect their lungs or spleens against aerosol-induced TB (52).

Consistent with the increased protection by DKO vaccine, spleens of mice collected at necropsy after rechallenge (day 120) contained higher numbers of CD4 and CD8 T cells secreting IFN-γ and IL-2 (**Figures 6C, D**).

### DKO vaccine induces both effector (T_EM_) and central (T_CM_) memory T cells contributing to robust protection against primary and reinfection tuberculosis in mice

In the mouse model, T cells play a major role in restricting *Mtb* growth and most TB vaccines induce and expand T-helper T cells. We therefore analyzed T_EM_ and T_CM_ cells in vaccinated and challenged mice using flow cytometry (gating strategy details and T cell populations shown in **Supplementary Figure 3**). Previous studies have shown that effector-T_EM_ T cells are generally CD62L^-^CCR7^-^CD44^hi^CD127^+/-^, whereas T_CM_ memory T cells are CD62L^+^CCR7^+/-^CD44^hi^ CD127^+^. We note that this classification is better suited for CD8 T cells since memory in CD4 T cells is more heterogeneous. T cells also were mapped based on their expression of CD44, which is an important marker for activation, migration, and homing. In addition, CD127 expression was measured because it helps in maintenance of memory.

**Figure 7:**
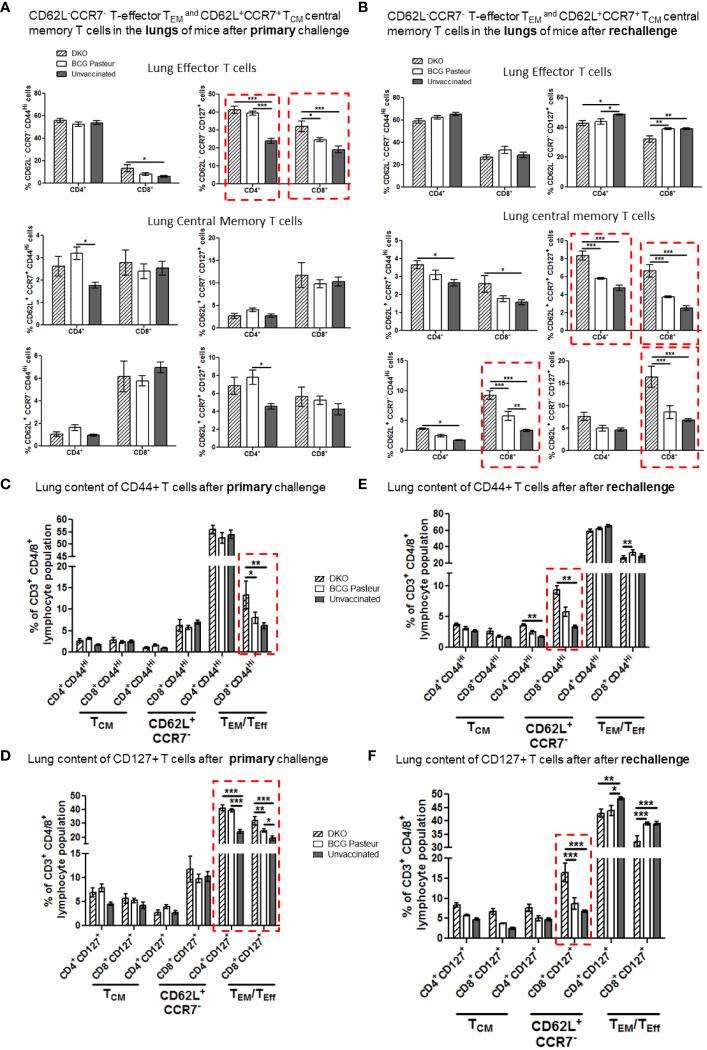
DKO vaccine induces robust T cell responses in the lungs of mice after primary aerosol challenge and re-challenge via expansion of effector and central memory T cells. **(A, B)** Lung T cells from mice (n=3) were isolated 3 weeks after primary challenge or after secondary challenge. Cells were stained to determine the numbers of effector (CD62L^-^ CD44^+^CCR7^-^CD127^+^ CD4/CD8 effector T cells (T_EM_) and CD62L^+^CD44^+^CCR7^-^/^+^CD127^+^ CD4/CD8 central memory T cells (T_CM_). **(C–F)** Proportion of T cells expressing CD44 and CD127 are shown (*,**p< 0.01; ***p< 0.009, one-way ANOVA with Dunnett’s’ *post hoc* test). Gating strategy details are shown in **Supplementary Figure 3**.

**Figure 8:**
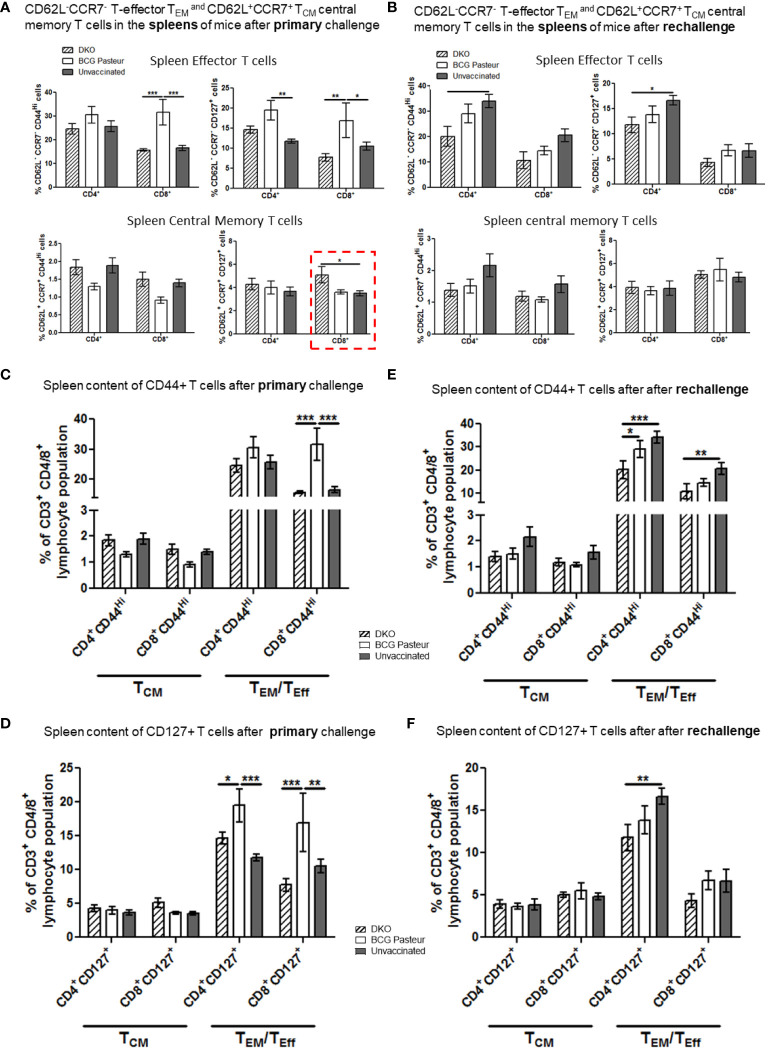
DKO vaccine induces robust T cell responses in the spleens of mice after primary aerosol challenge and re-challenge via expansion of effector and central memory T cells. **(A, B)** Splenic T cells from mice (n=3) were isolated 3 weeks after primary challenge or after secondary challenge. Cells were stained to determine the numbers of effector (CD62L^-^ CD44^+^CCR7^-^CD127^+^ CD4/CD8 effector T cells (T_EM_) and CD62L^+^CD44^+^CCR7^-^/^+^CD127^+^ CD4/CD8 central memory T cells (T_CM_). **(C–F)** Proportion of T cells expressing CD44 and CD127 are shown (*,**p< 0.01; ***p< 0.009, one-way ANOVA with Dunnett’s’ *post hoc* test).

Following subcutaneous vaccination of mice using BCG, the inguinal lymph nodes are the primary organs to process the vaccine and primed cells migrate into spleens and lungs, which are the target organ for *Mtb*. **Figures 7**, **8** show the T cell profiles of three organs following primary and rechallenge with *Mtb*.

**Figure 9:**
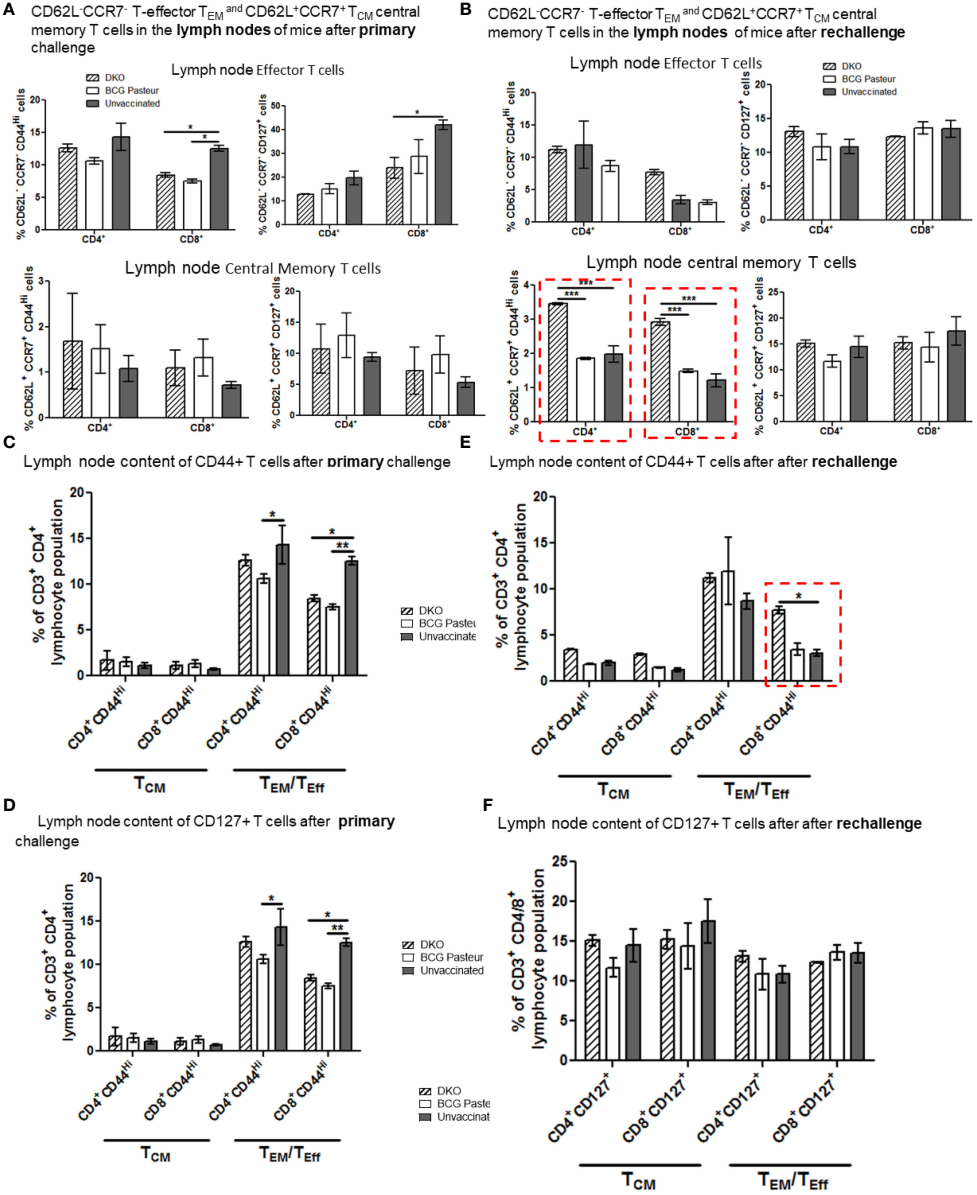
DKO vaccine induces robust T cell responses in the lymph nodes of mice after primary aerosol challenge and re-challenge via expansion of effector and central memory T cells. **(A, B)** Splenic T cells from mice (n=3) were isolated 3 weeks after primary challenge or after secondary challenge. Cells were stained to determine the numbers of effector (CD62L^-^ CD44^+^CCR7^-^CD127^+^ CD4/CD8 effector T cells (T_EM_) and CD62L^+^CD44^+^CCR7^-^/^+^CD127^+^ CD4/CD8 central memory T cells (T_CM_). **(C–F)** Proportion of T cells expressing CD44 and CD127 are shown (*,**p< 0.01; ***p< 0.009, one-way ANOVA with Dunnett’s’ *post hoc* test).

**Figure 7A** indicates that the lungs of DKO-vaccinated mice challenged with *Mtb* showed a robust expansion of T_EM_ compared to BCG-vaccinated mice, underscoring a potential role for T_EM_ in clearing *Mtb* organisms from the lungs. DKO-vaccinated mice subjected to *Mtb* re-challenge also showed a significant increase in T_CM_ along with an elevated level of T_EM_ (**Figures 7A, B**). when we analyzed lung T cells based upon CD44 and CD127 expression, DKO vaccinated mice showed increased CD44 and CD127 expressing T cells both after primary and rechallenge (**Figures 7C, D**). In contrast to lungs, spleens of DKO vaccinated mice showed less pronounced changes in T cell expression of memory markers (**Figure 8**). However, lymph nodes indicated a stronger expansion of T_CM_ after re-challenge (**Figure 9B**).

We propose that, compared to BCG vaccine, DKO likely induces protection against primary *Mtb* challenge mainly through a T_EM_ response, while its efficacy against *Mtb* re-challenge reflects the combined contributions of both T_CM_ and T_EM_. **Supplementary Figures 4**, **5** show T cells enriched for CD62L, CD44, CD127 and CCR7 in the organs of vaccinated mice. Further, **Supplementary Figure 6** illustrates that the DKO vaccinated but non challenged mice showed a stronger IFN-γ^+^ CD4 and CD8 T cells compared to BCG vaccine. We conclude that the *Mtb*-derived DKO vaccine generates a qualitatively superior T cell response against TB in mice compared to the BCG vaccine.

## Discussion

It is well established that BCG offers partial protection against TB in infants and children, but that its effectiveness diminishes with time. Thus, vaccines generating long-term protection are needed. Although BCG did not effectively reduce *Mtb* lung burden in rhesus non-human primates (NHPs) and cynomolgus NHPs (53), intravenous BCG was markedly protective (54). However, the safety of intravenous immunization in infants is unknown. Our previous studies demonstrated that the *Mtb* H37Rv-derived DKO mutant was highly immunogenic within MΦs *ex vivo* and exhibited attenuated growth in mice, similar to the BCG vaccine (8, 10) (**Supplementary Figure 1**). We hypothesized that DKO could serve as a more immunogenic candidate TB vaccine compared to BCG due to its retention of the RD1 region, which encodes six major immunogenic proteins, including *CFP-10* and *ESAT-6*. Unlike BCG, which lacks these immunogenic proteins, DKO can induce antibodies against these antigens, making it a promising candidate for further investigation (55). Here, we disrupted *sapM* and *fbpA* in *Mtb* to create a more attenuated vaccine, as the PL-fusion competent single gene mutants *fbpAKO* and *sapMKO* showed increased antigen processing in APCs (4, 8–10).

Others have also developed *Mtb*-derived vaccines, including the *faD26/phoP* double deletion mutant *MTB*VAC and the *Mtb*-*ΔsigH* vaccines, both of which contain the RD1 region and provide better protection against TB in macaques compared to BCG (56–59). This suggested that the RD1 region, which controls *Mtb* virulence (60–62), may be responsible for retaining immunogenicity due to its encoding of immunodominant antigens, such as ESAT-6, CFP-10, and TB10.4 (63, 64). Therefore, we tested a two-step hypothesis: first, that the RD1-encoded antigens increased the immunogenicity of DKO, and second, that *sapM* deletion enhanced antigen processing in APCs by facilitating autophagy and PL fusion.

We found that DKO phagosomes were enriched for PI-3P, LAMP1, Rab7, and vATPase (**Figure 1**). PL fusion begins with an accumulation of PI-3P on phagosomes which, in turn, tether RAB-GTPases like Rab5 and Rab7, resulting in PL fusion (65). We propose that the deletion of SapM acid phosphatase, which dephosphorylates PI-3P on the phagosomal membrane, enhances PL fusion in DKO-Supporting this, both wortmannin-mediated Inhibition of PI-3 kinase, which synthesizes PI3-P, and bafilomycin-dependent inhibition of the acidifying enzyme vATPase resulted in reduced antigen presentation (**Figure 3**). This suggests that DKO undergoes efficient PL fusion, resulting in increased antigen presentation. Consistent with this observation, DKO upregulated genes associated with autophagy induction in mouse and human MФs (**Figure 2**). Because SapM acid phosphatase also targets Rab7 during autophagy, we investigated whether DKO triggers autophagy. We found that DKO induces autophagy, leading to increased antigen presentation, which was reduced after autophagy knockdown (**Figure 3**). DKO is therefore unique in enhancing autophagy, likely due to the absence of SapM acid phosphatase.

Additional studies showed that blockade of lysosomal acidification, autophagy, and the inflammasome nearly abolished antigen presentation in DCs (**Figure 3**), suggesting that DKO induces robust antigen presentation in DCs through a combination of mechanisms involving autophagy-mediated degradation in lysosomes, the release of CTSB, inflammasome activation, and the release of IL-1β. This is further supported by the observation that DKO induced robust, caspase-dependent IL-1β production in APCs when compared to *sapMKO*, *fbpAKO*, *or Mtb*-H37Rv (**Figure 4**). Notably, DKO displayed reduced protection against TB in caspase-1/11-deficient mice. Importantly, virulent *Mtb* inhibits inflammasomes (39), and the DKO described herein activates both autophagy and inflammasomes, which enhances its immunogenicity in APCs.

We confirmed that the increased immunogenicity of DKO in *ex vivo* models was also reflected in mice. In both the NIH model of vaccine evaluation and a rechallenge model, DKO was more protective than the BCG vaccine in inducing a better expansion of T_CM_ and T_EM_ T cells (**Figures 6**, **7**). Notably, revaccination of C57Bl/6 mice with BCG did not confer protection to the lungs or spleen against TB, although a heterologous vaccination using subunit vaccines offered some degree of protection (52). Because DKO protected mice against TB better than BCG in the rechallenge model, we propose that the memory T cells induced by DKO last longer than those elicited by the BCG vaccine (**Figure 7**–**9**; **Supplementary Figures 4**-**6**). Given that childhood TB continues to occur in BCG-vaccinated infants, BCG-induced immunity appears to wane over time. We propose that boosting the primary BCG vaccine with an antigenically heterologous DKO may likely generate long-term protection due to its inclusion of RD1-encoded antigens. Therefore, additional gene disruptions may be necessary to render DKO completely safe for human use.

We conclude that *Mtb*-derived attenuated vaccines like DKO induce novel antigen processing mechanisms such as autophagy and inflammasome pathways in APCs which paves the way for new-generation vaccines for protection against TB and for boosting BCG vaccine.

## Methods

### Construction of DKO vaccine

This has been described in detail in our previous paper (8). APCs: Primary bone marrow-derived macrophages (MΦs) from wild-type and Caspase-1 knock out C57Bl/6J mice (4–8 weeks old, M/F, Harlan or Jackson animal providers, USA) were grown in Iscove’s medium with 10% FBS (IMDM) and 10 ng/mL GM-CSF. CD11c microbeads (Miltenyi Inc, USA;130–052-001) were used to deplete DCs from bone marrow cells cultured for 7 days. The CD11b+ CD11c- MΦs or CD11c bead-purified CD11c+ DCs were plated in GM-CSF-free medium and incubated overnight at 37°C. Subsequently, they were then infected with mycobacterial mutants and wild-type strains of *Mtb* (MOI=1), prepared as a single-cell suspension, for 4 h on a shaker at 37°C. Cells were then washed, and supernatants or cell lysates were collected for further analysis. Cell viability for both MΦ and DCs was assessed using trypan blue staining, with >90% viability confirmed both at the time of plating and at the end of experiments.

### *In vitro* presentation of Ag85B to BB7 CD4 T cells

MΦs and DCs were cultured in IMDM and used as monolayers in 24-well plates for IL-2 assays. The Ag85B p25 mouse epitope (spanning amino acids 241–256)-specific (BB7) T cell hybridoma was kindly provided by Drs. Cliff Harding and David Canaday, Case Western Reserve University, USA. Untreated or peptide-activated APCs were infected with various wild-type *Mtb* strains, its mutants, and *M. bovis* BCG (MOI=1). After 2 or 4 h, cells were washed and sonicated to obtain single cell CFU suspension, followed by washing monolayers with medium and overlaying with T cells (20:1 ratio). Supernatants were collected 4 or 24 h later, as indicated, and tested for IL-2 using sandwich ELISA.

### Mycobacteria

Wild-type BCG (wt-BCG Pasteur; ATCC #35734), *Mtb*-H37Rv (ATCC#27294), *Mtb*-Erdman (ATCC#35807), and *Mtb*-Erdman tagged with acriflavine resistance gene, all from the American Type Culture Collection (ATCC), were grown in Dubos’ broth and used after undergoing three washes in PBS. Cultured mycobacteria were routinely assessed for >90% viability using fluorescein diacetate staining (Invitrogen, USA). Recombinant BCG, including *gfp-*BCG, were grown in 7H9 broth containing kanamycin. Single-cell suspensions were used for APC infection, aerosol infection, or vaccination of mice. McFarland #1 matched suspensions were washed thoroughly using PBS containing 0.01% Tween 80 to remove debris, followed by sonication in PBS using a Bronson sonicator for 10 s, followed by centrifugation at 200g for 5 min to remove clumps. Resulting supernatants containing single CFUs were used for infection immediately after confirmation through acid-fast staining.

### Cytokine assay in APCs

Quantification of cytokines produced by MΦs and DCs was carried out by enzyme-linked immunosorbent assay (ELISA) using commercially available kits (Biolegend for mouse IL-12, TNF-α, IFN-β, and IL-1β). Supernatants were collected at indicated times after activation or blockade with Z-VAD-FMK/Y-VAD-FMK and other pharmacological agents, followed by infection with *Mtb*. Supernatants were filtered through a 0.22-µm filter (EMD Millipore, MA, USA) before they were titrated for cytokine levels according to the manufacturer’s protocol.

### *Mtb* growth assay in MФs

Primary mouse MΦs were lysed with 0.05% SDS at different timepoints post-*Mtb* infection with or without Z-VAD FMK/Y-VAD FMK and other pharmacological agents. Lysates were plated at serial 10-fold dilutions in PBS on 7H11 Middlebrook agar plates (Difco Laboratories, Surrey, UK). The agar plates were then incubated at 37°C for 3 weeks before counting CFUs. Data were expressed as log_10_ CFUs per million APCs.

### Induction of autophagy and *in situ* localization of autophagy markers

*Transfection of MΦs.* C57Bl/6J-derived MΦs were nucleotransfected with DNA encoding *rfp*-LC3 using the Amaxa kit with either vector control DNA or *rfp*-LC3 plasmid purified DNA. Transfected MΦs were treated with rapamycin as a positive control. Transfected MΦs were infected with *gfp*-BCG for 4 h, fixed and examined for colocalization using a laser confocal microscope (LCM). For peptide activation, MΦs were incubated with peptides as indicated, followed by infection with *gfp*-BCG and colocalization studies. During LCM analysis, a series of Z-sections were acquired and analyzed using 2D-deconvolution software. Percent colocalization was determined by counting colocalizing phagosomes within macrophages, averaging their number per 100 MΦs in quadruplicate slide chambers per mouse-derived bone marrow preparation three times per experiment. All scoring was blinded. The standard deviations were calculated from three independent experiments, each using MΦs from three mice. P values were determined for colocalization scores using t-tests.

### Mouse vaccine experiments

Two types of mouse vaccine experiments were conducted: a primary challenge and a re-challenge model (**Figures 5**, **6**). Both wild-type C57Bl/6J mice and Caspase-1 KO mice were used. Age- and sex-matched C57Bl/6J mice (6–8 weeks) were tested. Naïve or vaccinated (wild-type *Mtb* and mutant strains along with BCG) mice were given ~1x10^6^ CFUs subcutaneously in the hind leg. After vaccination, mice were aerosol-challenged with ~100 CFU virulent *Mtb*-Erdman using a Glas-Col (Indiana, USA) aerosol apparatus at indicated timepoints. Four weeks after the challenge or at indicated times, organs were harvested for CFUs, as previously described. Significant difference in the CFU counts was calculated using two-way ANOVA, as outlined below. We used five mice per vaccine strain, and three mice per timepoint for flow cytometry analysis. Challenge *Mtb* was differentiated from BCG vaccine by culturing organ homogenates in 7H11 agar containing Thiophene-2-carboxylic acid hydrazide (10 µg/mL), which inhibits BCG but not wild-type *Mtb*; or from DKO using 7H11 agar with acriflavine.

*Drug-induced clearance*: Ten mg/kg doses of INH and rifampin mixed in saline were given daily by gavage for three weeks, followed by rest as indicated. Organs were plated for CFUs on 7H11 agar to rule out persisting *Mtb*.

*P value for CFU counts in vaccinated vs. untreated control mice*: Five mice per group were vaccinated with BCG or left untreated as a control. Four weeks later, they were aerosol-challenged with *Mtb*. Mice were sacrificed and colony counts of *Mtb* were measured in the lungs. Data were plotted as log_10_ CFU per organ per mouse. The example above shows that such early CFU data are highly discriminative and predictive of survival. Importantly, the statistical power of these data is superior to survival data. Allowing for a statistical power of 0.8–0.9, and a usual variance of 0.1–0.2 log_10_ CFU, a reduction in the mean CFU values between saline controls and test groups of about 0.7 log_10_ CFU is significant when n=5 animals are used (one-way ANOVA used for p values).

### Pathology following Mtb infection

Our previous DKO vaccination studies showed no significant pathology in the organs compared to BCG vaccinated and Mtb vaccinated mice (8). Therefore, we monitored weight and health of mice until day 120 of sacrifice (**Supplementary Figure 6**) and vaccinated mice did not lose weight.

### Flow cytometry analysis of lung T cell and memory T cells

T cells from the infected lungs (post infection challenge) were quantified following established procedures. Briefly, three mice per dose-group were sacrificed. Lungs, spleen, and lymph nodes were teased in Iscove ‘s (IMDM) modification of Dulbecco medium and T cells were enriched from the resulting cell suspensions. Subsequently, T cells were stained for CD4 and CD8 markers, surface receptors, and intracellular IFN-γ and IL-2 followed by flow cytometric analysis. Results were reported as absolute numbers of T cells per organ after the initial organ cell count using trypan blue staining and acquiring a fixed number of cells. Lungs were processed with 1mg/mL collagenase and 1mg/mL elastase (Sigma Biologicals, USA) to break down the fibrous tissue material. Tissue was then passed through cell strainers and teased using frosted slides until a suspension of cells was obtained. Tissue was further treated with ACK lysis buffer (BioWhittaker, USA) to remove red blood cells. From one half of the spleen or lung tissue from each mouse, T cells were fractionated and analyzed for IFN-γ-secreting CD4 and CD8 T cells. We analyzed CD4 and CD8 T cell populations based on the expression of classic memory markers CD62L, CD44 and CD127. We determined the proportions of CD62L^-^ CCR7^−^ CD127^−^ CD44^+/−^ and CD62L^+^CCR7^+^ CD127^+^/CD44^+/−^ in the lungs, spleens and lymph nodes. We also mapped proportion of CD44 and CD127 because TB patients show reduced CD127 expression.

Flow staining was performed per BD Biosciences protocol. Cell events were collected using Beckman-Coulter-Gallios cytometer, and the cytokine profile was analyzed using FlowJo software (Tree Star, Ashland, OR). Graphs were plotted and analyzed using GraphPad Prism software version 8.

## Data availability statement

The original contributions presented in the study are included in the article/**Supplementary Material**. Further inquiries can be directed to the corresponding authors.

## Ethics statement

The animal study was approved by Institutional Animal Care and Use Committee, UTHSC-Houston, TX USA. The study was conducted in accordance with the local legislation and institutional requirements.

## Author contributions

AM: Writing – original draft, Visualization, Validation, Methodology, Investigation, Formal analysis, Data curation. AK: Writing – original draft, Methodology, Investigation, Data curation. VS: Data curation, Investigation, Methodology, Writing – original draft. SS: Writing – original draft, Methodology, Data curation. OG: Writing – original draft, Methodology, Data curation. KD: Writing – original draft, Methodology, Data curation. RV: Methodology, Writing – original draft, Data curation. SD: Writing – review & editing, Writing – original draft, Resources, Funding acquisition. CJ: Supervision, Methodology, Investigation, Data curation, Writing – review & editing, Writing – original draft, Resources, Funding acquisition, Conceptualization. EG: Writing – original draft, Methodology, Data curation.

## References

[1] Netea MG Joosten LA Latz E Mills KH Natoli G Stunnenberg HG. Trained immunity: A program of innate immune memory in health and disease. Science. (2016) 352:aaf1098. doi: 10.1126/science.aaf1098 27102489 PMC5087274

[2] Schrager LK Vekemens J Drager N Lewinsohn DM Olesen OF. The status of tuberculosis vaccine development. Lancet Infect Dis. (2020) 20:e28–37. doi: 10.1016/S1473-3099(19)30625-5 32014117

[3] Weerasuriya CK Clark RA White RG Harris RC. New tuberculosis vaccines: advances in clinical development and modelling. J Intern Med. (2020) 288:661–81. doi: 10.1111/joim.13197 33128834

[4] Singh CR Moulton RA Armitige LY Bidani A Snuggs M Dhandayuthapani S. Processing and presentation of a mycobacterial antigen 85B epitope by murine macrophages is dependent on the phagosomal acquisition of vacuolar proton ATPase and in *situ* activation of cathepsin D. J Immunol. (2006) 177:3250–9. doi: 10.4049/jimmunol.177.5.3250 16920965

[5] Jagannath C Lindsey DR Dhandayuthapani S Xu Y Hunter RL Jr. Eissa NT. Autophagy enhances the efficacy of BCG vaccine by increasing peptide presentation in mouse dendritic cells. Nat Med. (2009) 15:267–76. doi: 10.1038/nm.1928 19252503

[6] Behr MA Wilson MA Gill WP Salamon H Schoolnik GK Rane S. Comparative genomics of BCG vaccines by whole-genome DNA microarray. Science. (1999) 284:1520–3. doi: 10.1126/science.284.5419.1520 10348738

[7] Armitige LY Jagannath C Wanger AR Norris SJ. Disruption of the genes encoding antigen 85A and antigen 85B of *Mycobacterium tuberculosis* H37Rv: effect on growth in culture and in macrophages. Infect Immun. (2000) 68:767–78. doi: 10.1128/IAI.68.2.767-778.2000 PMC97204 10639445

[8] Saikolappan S Estrella J Sasindran SJ Khan A Armitige LY Jagannath C. The fbpA/sapM double knock out strain of *Mycobacterium tuberculosis* is highly attenuated and immunogenic in macrophages. PloS One. (2012) 7:e36198. doi: 10.1371/journal.pone.0036198 22574140 PMC3344844

[9] Copenhaver RH Sepulveda E Armitige LY Actor JK Wanger A Norris SJ. A mutant of *Mycobacterium tuberculosis* H37Rv that lacks expression of antigen 85A is attenuated in mice but retains vaccinogenic potential. Infect Immun. (2004) 72:7084–95. doi: 10.1128/IAI.72.12.7084-7095.2004 PMC529100 15557632

[10] Katti MK Dai G Armitige LY Rivera Marrero C Daniel S Singh CR. The Delta fbpA mutant derived from *Mycobacterium tuberculosis* H37Rv has an enhanced susceptibility to intracellular antimicrobial oxidative mechanisms, undergoes limited phagosome maturation and activates macrophages and dendritic cells. Cell Microbiol. (2008) 10:1286–303. doi: 10.1111/j.1462-5822.2008.01126.x PMC3668688 18248626

[11] Chai Q Wang L Liu CH Ge B. New insights into the evasion of host innate immunity by *Mycobacterium tuberculosis* . Cell Mol Immunol. (2020) 17:901–13. doi: 10.1038/s41423-020-0502-z PMC7608469 32728204

[12] Vergne I Chua J Lee HH Lucas M Belisle J Deretic V. Mechanism of phagolysosome biogenesis block by viable *Mycobacterium tuberculosis* . Proc Natl Acad Sci U.S.A. (2005) 102:4033–8. doi: 10.1073/pnas.0409716102 PMC554822 15753315

[13] Ramachandra L Smialek JL Shank SS Convery M Boom WH Harding CV. Phagosomal processing of *Mycobacterium tuberculosis* antigen 85B is modulated independently of mycobacterial viability and phagosome maturation. Infect Immun. (2005) 73:1097–105. doi: 10.1128/IAI.73.2.1097-1105.2005 PMC547092 15664953

[14] Ramachandra L Noss E Boom WH Harding CV. Processing of Mycobacterium tuberculosis antigen 85B involves intraphagosomal formation of peptide-major histocompatibility complex II complexes and is inhibited by live bacilli that decrease phagosome maturation. J Exp Med. (2001) 194:1421–32. doi: 10.1084/jem.194.10.1421 PMC2193679 11714749

[15] Perez-Montesinos G Lopez-Ortega O Piedra-Reyes J Bonifaz LC Moreno J. Dynamic changes in the intracellular association of selected rab small GTPases with MHC class II and DM during dendritic cell maturation. Front Immunol. (2017) 8:340. doi: 10.3389/fimmu.2017.00340 28396666 PMC5367080

[16] Deretic V Via LE Fratti RA Deretic D. Mycobacterial phagosome maturation, rab proteins, and intracellular trafficking. Electrophoresis. (1997) 18:2542–7. doi: 10.1002/elps.1150181409 9527483

[17] Roberts EA Chua J Kyei GB Deretic V. Higher order Rab programming in phagolysosome biogenesis. J Cell Biol. (2006) 174:923–9. doi: 10.1083/jcb.200603026 PMC2064384 16982798

[18] Via LE Deretic D Ulmer RJ Hibler NS Huber LA Deretic V. Arrest of mycobacterial phagosome maturation is caused by a block in vesicle fusion between stages controlled by rab5 and rab7. J Biol Chem. (1997) 272:13326–31. doi: 10.1074/jbc.272.20.13326 9148954

[19] Via LE Fratti RA McFalone M Pagan-Ramos E Deretic D Deretic V. Effects of cytokines on mycobacterial phagosome maturation. J Cell Sci. (1998) 111:897–905. doi: 10.1242/jcs.111.7.897 9490634

[20] Deretic V. Autophagy as an immune defense mechanism. Curr Opin Immunol. (2006) 18:375–82. doi: 10.1016/j.coi.2006.05.019 16782319

[21] Deretic V Delgado M Vergne I Master S De Haro S Ponpuak M. Autophagy in immunity against *mycobacterium tuberculosis*: a model system to dissect immunological roles of autophagy. Curr Top Microbiol Immunol. (2009) 335:169–88. doi: 10.1007/978-3-642-00302-8_8 PMC2788935 19802565

[22] Galluzzi L Baehrecke EH Ballabio A Boya P Bravo-San Pedro JM Cecconi F. Molecular definitions of autophagy and related processes. EMBO J. (2017) 36:1811–36. doi: 10.15252/embj.201796697 PMC5494474 28596378

[23] Khan A Sayedahmed EE Singh VK Mishra A Dorta-Estremera S Nookala S. A recombinant bovine adenoviral mucosal vaccine expressing mycobacterial antigen-85B generates robust protection against tuberculosis in mice. Cell Rep Med. (2021) 2:100372. doi: 10.1016/j.xcrm.2021.100372 34467249 PMC8385328

[24] Matta SK Kumar D. Hypoxia and classical activation limits *Mycobacterium tuberculosis* survival by Akt-dependent glycolytic shift in macrophages. Cell Death Discovery. (2016) 2:16022. doi: 10.1038/cddiscovery.2016.22 27551515 PMC4979487

[25] Hu D Wu J Wang W Mu M Zhao R Xu X. Autophagy regulation revealed by SapM-induced block of autophagosome-lysosome fusion via binding RAB7. Biochem Biophys Res Commun. (2015) 461:401–7. doi: 10.1016/j.bbrc.2015.04.051 25896765

[26] Shibutani ST Saitoh T Nowag H Munz C Yoshimori T. Autophagy and autophagy-related proteins in the immune system. Nat Immunol. (2015) 16:1014–24. doi: 10.1038/ni.3273 26382870

[27] Modica G Lefrancois S. Post-translational modifications: How to modulate Rab7 functions. Small GTPases. (2020) 11:167–73. doi: 1080/21541248.2017.1387686 10.1080/21541248.2017.1387686 PMC7549697 29099291

[28] Refai A Gritli S Barbouche MR Essafi M. *Mycobacterium tuberculosis* virulent factor ESAT-6 drives macrophage differentiation toward the pro-inflammatory M1 phenotype and subsequently switches it to the anti-inflammatory M2 phenotype. Front Cell Infect Microbiol. (2018) 8:327. doi: 10.3389/fcimb.2018.00327 30283745 PMC6157333

[29] Kim BH Shenoy AR Kumar P Das R Tiwari S MacMicking JD. A family of IFN-gamma-inducible 65-kD GTPases protects against bacterial infection. Science. (2011) 332:717–21. doi: 10.1126/science.1201711 21551061

[30] Zulauf KE Sullivan JT Braunstein M. The SecA2 pathway of Mycobacterium tuberculosis exports effectors that work in concert to arrest phagosome and autophagosome maturation. PloS Pathog. (2018) 14:e1007011. doi: 10.1371/journal.ppat.1007011 29709019 PMC5945054

[31] Jagannath C Bakhru P. Rapamycin-induced enhancement of vaccine efficacy in mice. Methods Mol Biol. (2012) 821:295–303. doi: 10.1007/978-1-61779-430-8_18 22125073 PMC3387998

[32] Harding CV. Class II antigen processing: analysis of compartments and functions. Crit Rev Immunol. (1996) 16:13–29. doi: 10.1615/CritRevImmunol.v16.i1 8809471

[33] Chen M Hong MJ Sun H Wang L Shi X Gilbert BE. Essential role for autophagy in the maintenance of immunological memory against influenza infection. Nat Med. (2014) 20:503–10. doi: 10.1038/nm.3521 PMC4066663 24747745

[34] Schenk M Fabri M Krutzik SR Lee DJ Vu DM Sieling PA. Interleukin-1beta triggers the differentiation of macrophages with enhanced capacity to present mycobacterial antigen to T cells. Immunology. (2014) 141:174–80. doi: 10.1111/imm.12167 PMC3904238 24032597

[35] Germic N Frangez Z Yousefi S Simon HU. Regulation of the innate immune system by autophagy: monocytes, macrophages, dendritic cells and antigen presentation. Cell Death Differ. (2019) 26:715–27. doi: 10.1038/s41418-019-0297-6 PMC6460400 30737475

[36] Biasizzo M Kopitar-Jerala N. Interplay between NLRP3 inflammasome and autophagy. Front Immunol. (2020) 11:591803. doi: 10.3389/fimmu.2020.591803 33163006 PMC7583715

[37] Amaral EP Riteau N Moayeri M Maier N Mayer-Barber KD Pereira RM. Lysosomal cathepsin release is required for NLRP3-inflammasome activation by mycobacterium tuberculosis in infected macrophages. Front Immunol. (2018) 9:1427. doi: 10.3389/fimmu.2018.01427 29977244 PMC6021483

[38] Sousa J Ca B Maceiras AR Simoes-Costa L Fonseca KL Fernandes AI. *Mycobacterium tuberculosis* associated with severe tuberculosis evades cytosolic surveillance systems and modulates IL-1beta production. Nat Commun. (2020) 11:1949. doi: 10.1038/s41467-020-15832-6 32327653 PMC7181847

[39] Rastogi S Ellinwood S Augenstreich J Mayer-Barber KD Briken V. *Mycobacterium tuberculosis* inhibits the NLRP3 inflammasome activation via its phosphokinase PknF. PloS Pathog. (2021) 17:e1009712. doi: 10.1371/journal.ppat.1009712 34324582 PMC8321130

[40] Krakauer T. Inflammasomes, autophagy, and cell death: the trinity of innate host defense against intracellular bacteria. Mediators Inflammation. (2019) 2019:2471215. doi: 10.1155/2019/2471215 PMC6341260 30728749

[41] Yao Y Chen S Cao M Fan X Yang T Huang Y. Antigen-specific CD8(+) T cell feedback activates NLRP3 inflammasome in antigen-presenting cells through perforin. Nat Commun. (2017) 8:15402. doi: 10.1038/ncomms15402 28537251 PMC5458103

[42] Lupfer C Kanneganti TD. Unsolved mysteries in NLR biology. Front Immunol. (2013) 4:285. doi: 10.3389/fimmu.2013.00285 24062750 PMC3775000

[43] Saiga H Nieuwenhuizen N Gengenbacher M Koehler AB Schuerer S Moura-Alves P. The recombinant BCG deltaureC::hly vaccine targets the AIM2 inflammasome to induce autophagy and inflammation. J Infect Dis. (2015) 211:1831–41. doi: 10.1093/infdis/jiu675 25505299

[44] Kim JS Kim WS Choi HG Jang B Lee K Park JH. *Mycobacterium tuberculosis* RpfB drives Th1-type T cell immunity via a TLR4-dependent activation of dendritic cells. J Leukoc Biol. (2013) 94:733–49. doi: 10.1189/jlb.0912435 23825389

[45] Kim WS Kim JS Cha SB Kim H Kwon KW Kim SJ. Mycobacterium tuberculosis Rv3628 drives Th1-type T cell immunity via TLR2-mediated activation of dendritic cells and displays vaccine potential against the hyper-virulent Beijing K strain. Oncotarget. (2016) 7:24962–82. doi: 10.18632/oncotarget.8771 PMC5041883 27097115

[46] Kim WS Kim JS Cha SB Kim SJ Kim H Kwon KW. Mycobacterium tuberculosis PE27 activates dendritic cells and contributes to Th1-polarized memory immune responses during in vivo infection. Immunobiology. (2016) 221:440–53. doi: 10.1016/j.imbio.2015.11.006 26655143

[47] Qu Y Ramachandra L Mohr S Franchi L Harding CV Nunez G. P2X7 receptor-stimulated secretion of MHC class II-containing exosomes requires the ASC/NLRP3 inflammasome but is independent of caspase-1. J Immunol. (2009) 182:5052–62. doi: 10.4049/jimmunol.0802968 PMC2768485 19342685

[48] Ramachandra L Noss E Boom WH Harding CV. Phagocytic processing of antigens for presentation by class II major histocompatibility complex molecules. Cell Microbiol. (1999) 1:205–14. doi: 10.1046/j.1462-5822.1999.00026.x 11207553

[49] Orme IM. The achilles heel of BCG. Tuberculosis (Edinb). (2010) 90:329–32. doi: 10.1016/j.tube.2010.06.002 20659816

[50] Repique CJ Li A Collins FM Morris SL. DNA immunization in a mouse model of latent tuberculosis: effect of DNA vaccination on reactivation of disease and on reinfection with a secondary challenge. Infect Immun. (2002) 70:3318–23. doi: 10.1128/IAI.70.7.3318-3323.2002 PMC128037 12065468

[51] Henao-Tamayo M Obregon-Henao A Ordway DJ Shang S Duncan CG Orme IM. A mouse model of tuberculosis reinfection. Tuberculosis (Edinb). (2012) 92:211–7. doi: 10.1016/j.tube.2012.02.008 PMC12543033 22429719

[52] Tiwari S Dutt TS Chen B Chen M Kim J Dai AZ. BCG-Prime and boost with Esx-5 secretion system deletion mutant leads to better protection against clinical strains of Mycobacterium tuberculosis. Vaccine. (2020) 38:7156–65. doi: 10.1016/j.vaccine.2020.08.004 PMC7755135 32978002

[53] Langermans JA Andersen P van Soolingen D Vervenne RA Frost PA van der Laan T. Divergent effect of bacillus Calmette-Guerin (BCG) vaccination on *Mycobacterium tuberculosis* infection in highly related macaque species: implications for primate models in tuberculosis vaccine research. Proc Natl Acad Sci U.S.A. (2001) 98:11497–502. doi: 10.1073/pnas.201404898 PMC58758 11562492

[54] Darrah PA Zeppa JJ Maiello P Hackney JA Wadsworth MH 2nd Hughes TK. Prevention of tuberculosis in macaques after intravenous BCG immunization. Nature. (2020) 577:95–102. doi: 10.1038/s41586-019-1817-8 31894150 PMC7015856

[55] Brusasca PN Colangeli R Lyashchenko KP Zhao X Vogelstein M Spencer JS. Immunological characterization of antigens encoded by the RD1 region of the *Mycobacterium tuberculosis* genome. Scand J Immunol. (2001) 54:448–52. doi: 10.1046/j.1365-3083.2001.00975.x 11696195

[56] Kaushal D Foreman TW Gautam US Alvarez X Adekambi T Rangel-Moreno J. Mucosal vaccination with attenuated *Mycobacterium tuberculosis* induces strong central memory responses and protects against tuberculosis. Nat Commun. (2015) 6:8533. doi: 10.1038/ncomms9533 26460802 PMC4608260

[57] White AD Sibley L Sarfas C Morrison A Gullick J Clark S. MTBVAC vaccination protects rhesus macaques against aerosol challenge with M. tuberculosis and induces immune signatures analogous to those observed in clinical studies. NPJ Vaccines. (2021) 6:4. doi: 10.1038/s41541-020-00262-8 33397991 PMC7782851

[58] Gonzalo-Asensio J Marinova D Martin C Aguilo N. MTBVAC: attenuating the human pathogen of tuberculosis (TB) toward a promising vaccine against the TB epidemic. Front Immunol. (2017) 8:1803. doi: 10.3389/fimmu.2017.01803 29326700 PMC5736532

[59] Perez I Uranga S Sayes F Frigui W Samper S Arbues A. Live attenuated TB vaccines representing the three modern Mycobacterium tuberculosis lineages reveal that the Euro-American genetic background confers optimal vaccine potential. EBioMedicine. (2020) 55:102761. doi: 10.1016/j.ebiom.2020.102761 32361249 PMC7195525

[60] Pym AS Brodin P Brosch R Huerre M Cole ST. Loss of RD1 contributed to the attenuation of the live tuberculosis vaccines *Mycobacterium bovis* BCG and Mycobacterium microti. Mol Microbiol. (2002) 46:709–17. doi: 10.1046/j.1365-2958.2002.03237.x 12410828

[61] Brodin P Majlessi L Marsollier L de Jonge MI Bottai D Demangel C. Dissection of ESAT-6 system 1 of *Mycobacterium tuberculosis* and impact on immunogenicity and virulence. Infect Immun. (2006) 74:88–98. doi: 10.1128/IAI.74.1.88-98.2006 16368961 PMC1346617

[62] Bottai D Majlessi L Simeone R Frigui W Laurent C Lenormand P. ESAT-6 secretion-independent impact of ESX-1 genes espF and espG1 on virulence of Mycobacterium tuberculosis. J Infect Dis. (2011) 203:1155–64. doi: 10.1093/infdis/jiq089 21196469

[63] Etna MP Giacomini E Pardini M Severa M Bottai D Cruciani M. Impact of Mycobacterium tuberculosis RD1-locus on human primary dendritic cell immune functions. Sci Rep. (2015) 5:17078. doi: 10.1038/srep17078 26602835 PMC4658526

[64] Mustafa AS. Immunological characterization of proteins expressed by genes located in *mycobacterium tuberculosis*-specific genomic regions encoding the ESAT6-like proteins. Vaccines (Basel). (2021) 9(1):27. doi: 10.3390/vaccines9010027 33430286 PMC7825740

[65] Langemeyer L Frohlich F Ungermann C. Rab GTPase function in endosome and lysosome biogenesis. Trends Cell Biol. (2018) 28:957–70. doi: 10.1016/j.tcb.2018.06.007 30025982

