# Supplementary material for: The ΔfbpAΔsapM candidate vaccine derived from Mycobacterium tuberculosis H37Rv is markedly immunogenic in macrophages and induces robust immunity to tuberculosis in mice
Source: Front Immunol. 2024 Jun 21;15:1321657. doi: 10.3389/fimmu.2024.1321657 (PMC11224292; doi:10.3389/fimmu.2024.1321657)
Supplement: Supplementary file 1 [file DataSheet_1.docx]

**SUPPLEMENTAL FIGURES**

**The *ΔfbpAΔsapM* candidate vaccine derived from *Mycobacterium tuberculosis* H37Rv is markedly immunogenic in macrophages and induces robust immunity to tuberculosis in mice**

*Running title: Tuberculosis vaccine activating autophagy and inflammasome*

Abhishek Mishra^1#^, Arshad Khan^1#^, Vipul Kumar Singh^1^, Emily Glyde^1^,, Sankaralingam Saikolappan^2^, Omar Garnica^2^, Kishore Das^2^, Raja Veerapandian^2^, Subramanian Dhandayuthapani ^2^* and Chinnaswamy Jagannath^1#^*

^1^ Department of Pathology and Genomic Medicine, Houston Methodist Research Institute, Weill-Cornell Medicine, Houston, Texas

^2^ Department of Molecular and Translational Medicine, Texas Tech University Health Sciences Center, El Paso, Texas

#These authors contributed equally to this work

**Correspondence*

Chinnaswamy Jagannath

[cjagannath@houstonmethodist.org](mailto:cjagannath@houstonmethodist.org)

Subramanian Dhandayuthapani

[S.Dhandayuthapani@ttuhsc.edu](mailto:S.Dhandayuthapani@ttuhsc.edu)

**Supplemental Figure-1:** Growth curves of DKO in macrophages and mice related to text.

**
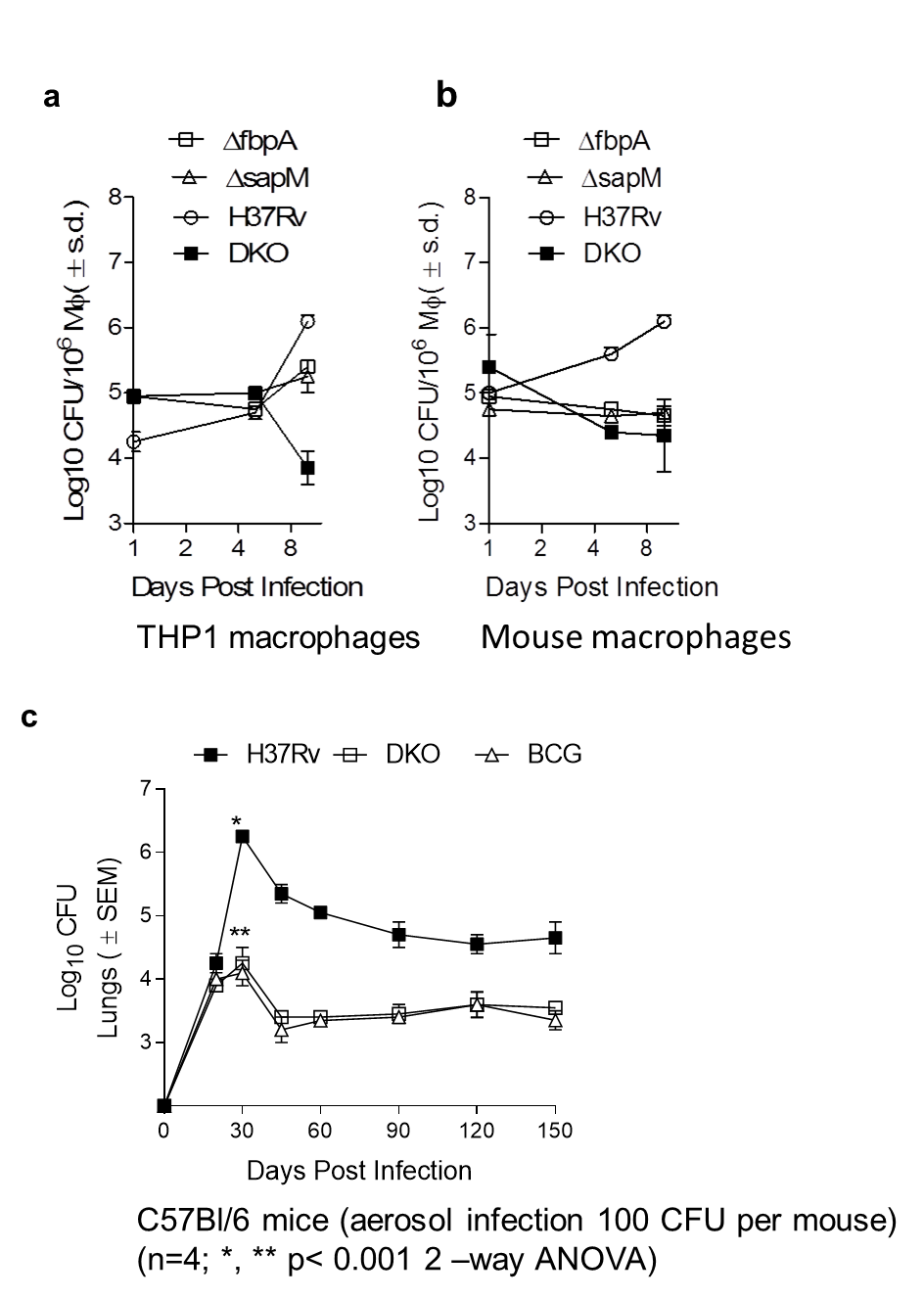
**

**Supplemental Figure-2:** (**a-b)** Wild type MФs and DCs were pretreated with pan-caspase specific inhibitor ZVAD-fmk and caspase-1 specific inhibitor YVAD-fmk (40 µM each) and supernatants tested for IL-1β at 18 hr. after mycobacterial infection. ( * p< 0.007 **p< 0.003, one-way ANOVA with Tukey’s post hoc test). NOTE: IL-1β levels are higher than shown in Fig.3; however, these are C57Bl/6J mice and are different from caspase KO mice of Fig.3.


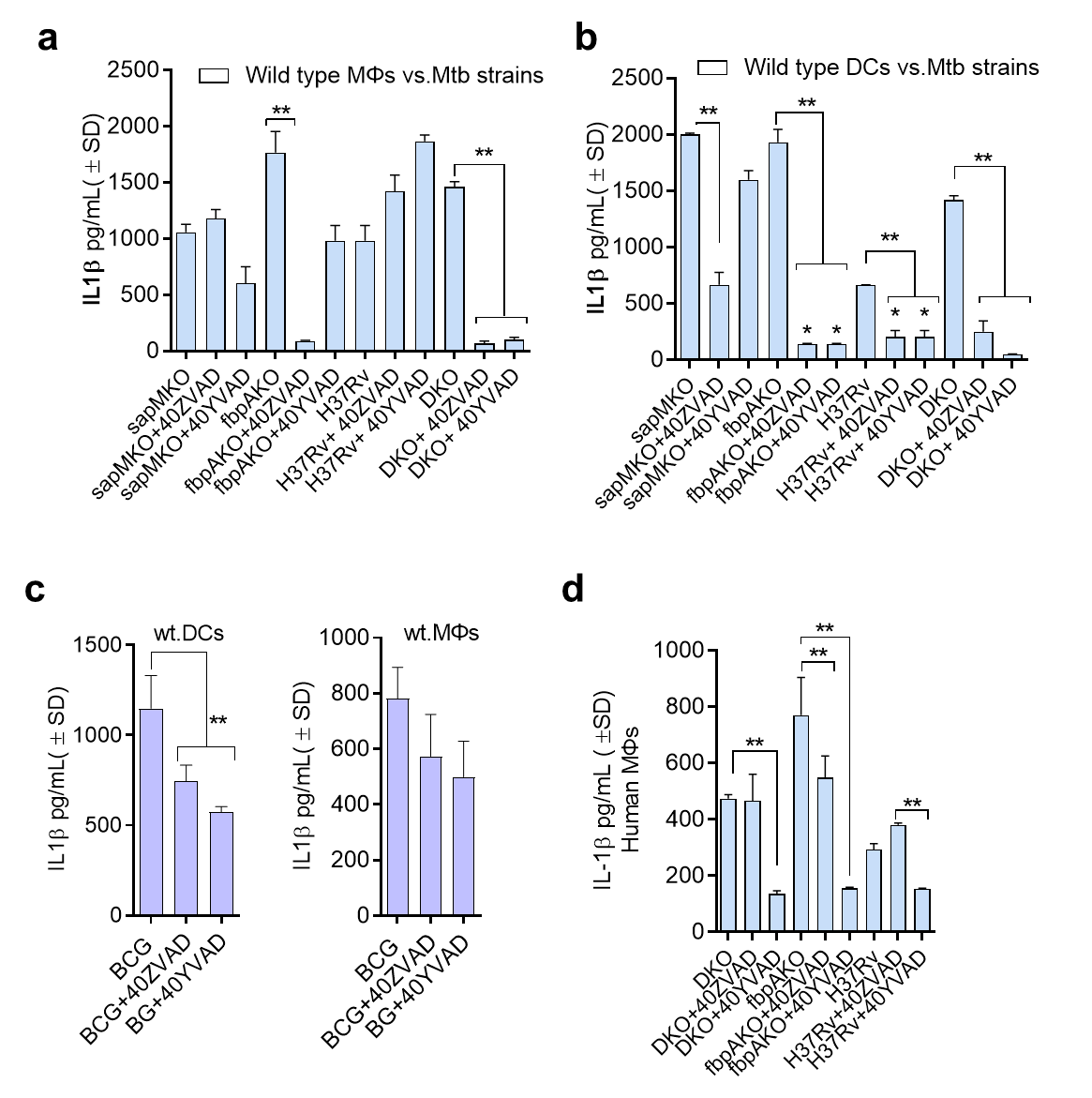


**Supplemental Figure-3:** Gating strategy for Flow cytometry analysis related to Fig.7.

**
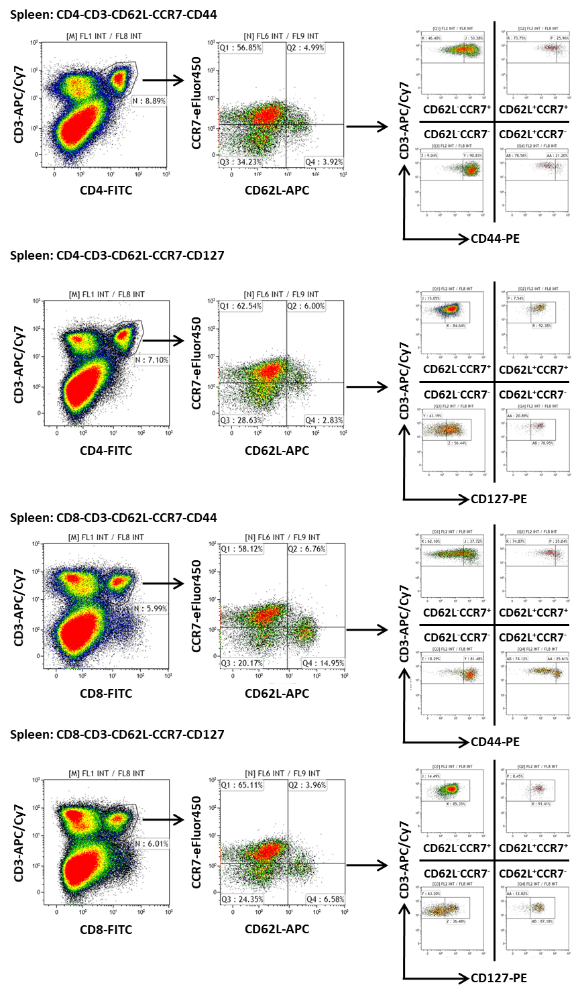

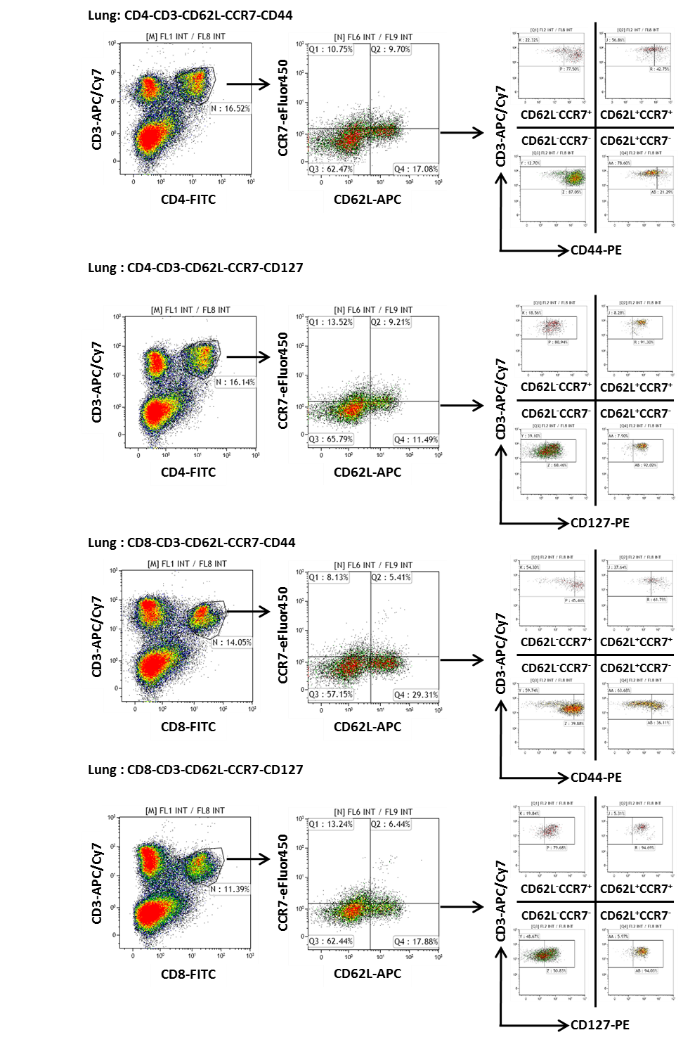
Supplemental Figure-4:** T cell profiles of mice post Mtb challenge (pretreatment) related to Fig.7-9. Distribution of CD62L, CD44, CD127 and CCR7 post-primary challenge with tuberculosis(p values as in text).


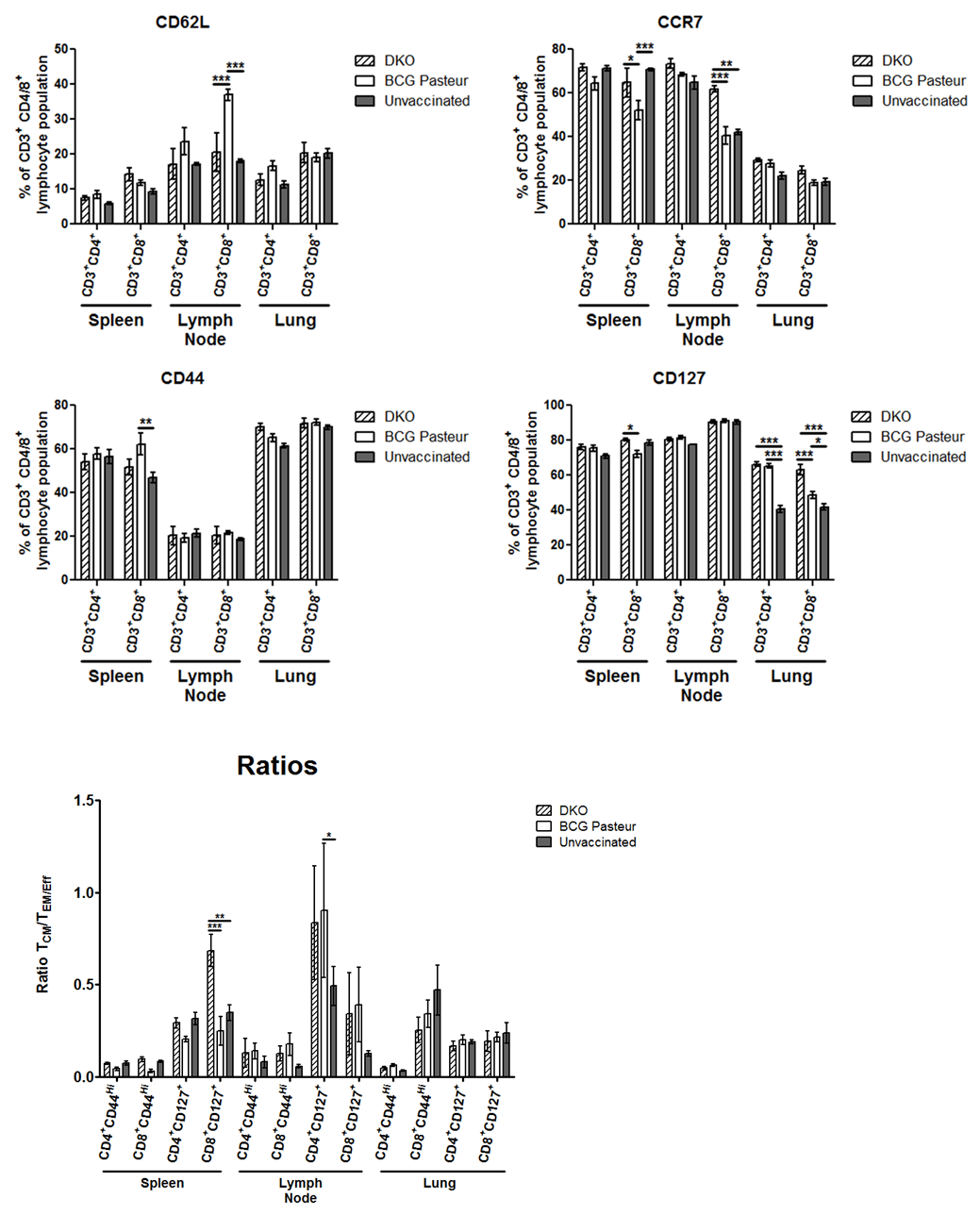


**Supplemental Figure-5:** T cell profiles of mice post Mtb challenge (pretreatment) related to Fig.7-9. Distribution of CD62L, CD44, CD127 and CCR7 post-secondary challenge with tuberculosis (p values as in text).


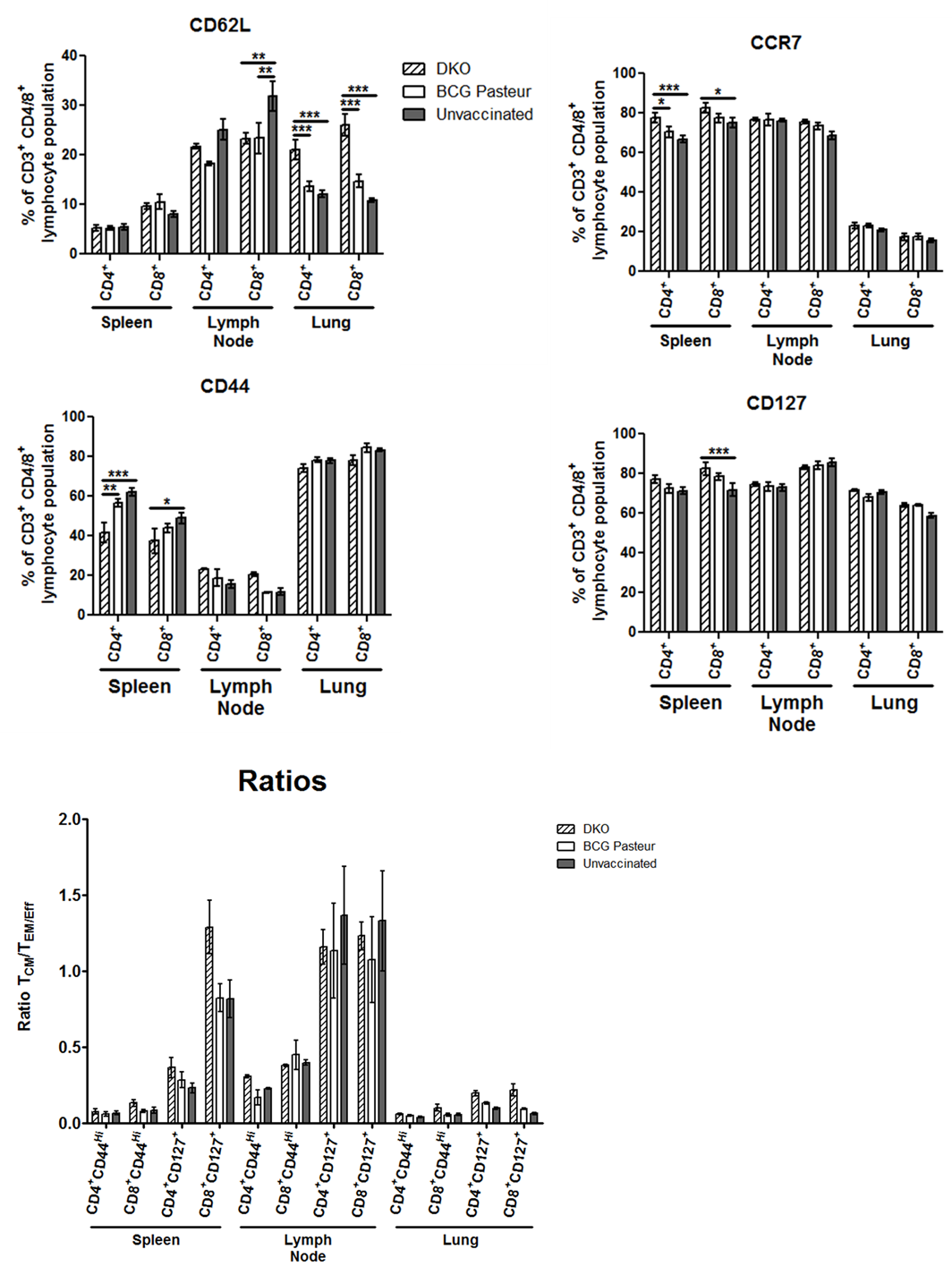


**Supplemental Figure-6:** Weight and health monitoring of mice after vaccination Day 120 Necropsy. Compared to unvaccinated healthy unchallenged mice, Mtb infected mice showed decrease in weight by day 120. Neither BCG nor DKO vaccinated mice showed a significant decrease in weight.
